# Characterization of a multipurpose NS3 surface patch coordinating HCV replicase assembly and virion morphogenesis

**DOI:** 10.1371/journal.ppat.1010895

**Published:** 2022-10-10

**Authors:** Olaf Isken, Minh Tu Pham, Hella Schwanke, Felicia Schlotthauer, Ralf Bartenschlager, Norbert Tautz

**Affiliations:** 1 Institute of Virology and Cell Biology, University of Luebeck, Luebeck, Germany; 2 Department of Infectious Diseases, Molecular Virology, Heidelberg University, Heidelberg, Germany; 3 German Center for Infection Research, Heidelberg partner site, Heidelberg, Germany; University of California, San Diego, UNITED STATES

## Abstract

The hepatitis C virus (HCV) life cycle is highly regulated and characterized by a step-wise succession of interactions between viral and host cell proteins resulting in the assembly of macromolecular complexes, which catalyse genome replication and/or virus production. Non-structural (NS) protein 3, comprising a protease and a helicase domain, is involved in orchestrating these processes by undergoing protein interactions in a temporal fashion. Recently, we identified a multifunctional NS3 protease surface patch promoting pivotal protein-protein interactions required for early steps of the HCV life cycle, including NS3-mediated NS2 protease activation and interactions required for replicase assembly. In this work, we extend this knowledge by identifying further NS3 surface determinants important for NS5A hyperphosphorylation, replicase assembly or virion morphogenesis, which map to protease and helicase domain and form a contiguous NS3 surface area. Functional interrogation led to the identification of phylogenetically conserved amino acid positions exerting a critical function in virion production without affecting RNA replication. These findings illustrate that NS3 uses a multipurpose protein surface to orchestrate the step-wise assembly of functionally distinct multiprotein complexes. Taken together, our data provide a basis to dissect the temporal formation of viral multiprotein complexes required for the individual steps of the HCV life cycle.

## Author summary

HCV genome replication and virion morphogenesis take place in dedicated cellular membrane compartments that serve as assembly sites of specialized protein complexes executing these processes. Maturation of these macromolecular protein complexes requires a step-wise succession of protein-protein interactions that must be tightly controlled in a spatio-temporal manner to avoid the formation of non-functional protein complexes. NS3 has a pivotal role in this temporal regulation by providing multipurpose surface areas for the assembly of functionally distinct protein complexes. By interrogating these NS3 surface areas, we show that a set of phylogenetically conserved NS3 residues forms a continuous surface patch across protease and helicase domain that is critical for NS5A hyperphosphorylation, a feature shown to be important for replicase formation to support HCV RNA replication. Furthermore, a helicase residue in close proximity to these residues is pivotal for virion morphogenesis without affecting genome replication. We propose that NS3 provides distinct surface areas to regulate, in a temporal and spatial fashion, the assembly of various, dedicated protein complexes during the different steps of the hepaciviral life cycle that are crucial for replicase assembly and virion formation.

## Introduction

Hepatitis C virus (HCV) belongs to the family *Flaviviridae* and is an important cause of chronic liver disease worldwide [1]. The RNA genome of positive polarity comprises a 5’-untranslated region (UTR), a single open reading frame (ORF) that encodes structural and non-structural (NS) viral proteins and a 3’ UTR. Translation of the viral ORF is initiated in a cap-independent mode and yields a single polyprotein that is co- and post-translationally processed into the mature proteins by host enzymes and two viral proteases encoded by the NS2-NS3 and NS3-4A proteins, respectively [2]. The host signal peptidases mediate cleavages at the junctions of core/E1, E1/E2, E2/p7 and p7/NS2 [3–5]. Processing of the NS2-NS3 precursor protein is mediated by an autoprotease located in NS2, which is activated by NS3 [6,7]. All cleavages downstream of NS3 are mediated by the NS3/NS4A serine protease complex [8,9]. It is a chymotrypsin-like serine protease residing in the N-terminal part of NS3 and requires NS4A as cofactor essential for full proteolytic activity [8,10,11]. NS3 is a multifunctional protein and harbors downstream of the protease domain a dedicated helicase domain with ATPase and helicase activities, which are both essential for RNA replication. The NS4A protease cofactor is anchoring the NS3/NS4A complex to cellular membranes by its N-terminal transmembrane domain. Dimerization of NS4A *via* this transmembrane region is required for HCV RNA replication and virus particle assembly [12,13]. NS4B is a membrane protein involved in the assembly of the viral replication complex (RC) [14–19]. NS5A is a multi-functional phosphoprotein that exerts essential roles throughout the viral life cycle [20–25]. NS5B is the RNA-dependent RNA polymerase (RdRp) [26–28]. While NS2 is dispensable for RNA replication it is essential for virus assembly. The polyprotein region NS3-NS5B is necessary and sufficient for formation of the membrane-associated RNA replication complex [19,29].

HCV genome replication is regulated by a step-wise action of dedicated macromolecular complexes assembled by specific interactions between viral and host proteins in association with cellular membranes, creating a specialized environment for the different steps of the HCV life cycle [30]. Those complexes catalyse important steps such as polyprotein processing, genome replication and infectious virus particle production, which often take place *in cis* on the same viral RNA molecule, thereby requiring tight control in time and space. A peculiarity of the *Flaviviridae* family members is that non-structural proteins are critical for both genome replication and virion morphogenesis [13,31–40]. In the case of HCV, the NS3 protein is, due to its multifunctional nature, exquisitely suited to orchestrate these processes in a temporal fashion [33,41–44]. During HCV genome replication, the NS3 protein is responsible for different tasks such as polyprotein cleavages, unwinding of nucleic acids, displacement of proteins bound to nucleic acids, and packaging of the RNA genome to form infectious viral particles [41–43,45,46]. NS3 exerts these activities by engaging in an array of intra- and intermolecular interactions within different multiprotein complexes [33,36,40,43,47–52]. By doing so, NS3 undergoes, in complex with NS4A, interactions with viral proteins such as core, NS2, NS4B and NS5A [13,40,43,51,53,54].

Recently, it was demonstrated that interactions between conserved hydrophobic NS3 surface residues on the protease domain and yet uncharacterized NS2 determinants of newly translated NS2-NS3 precursor proteins promote NS2 protease activation by NS3 for efficient NS2-NS3 cleavage in HCV and other non-human mammalian hepaciviruses [43,55]. Upon NS3 release, this hydrophobic NS3 surface patch subsequently supports protein interactions of NS3 that are resulting in HCV NS5A hyperphosphorylation and replicase assembly [6,43,56]. Accordingly, the step-wise use of this NS3 surface patch offers an explanation why NS2-NS3 cleavage and NS3 release is a prerequisite for functional replicase assembly [41,56]. Together, these observations illustrate how viruses are controlling the assembly of functionally different complexes by mutually exclusive protein-protein interactions involving an overlapping set of proteins.

To define further contributing factors for these sequential protein complex formations, here we extended our previous analysis of NS3 surface determinants on the protease and helicase domain to identify additional NS3 surface residues critical for RC assembly or virion morphogenesis. Interestingly, determinants critical for replicase assembly were mapped to both the protease or helicase domain and form together with our recently identified NS3 surface residues a contiguous NS3 surface area linking the protease and helicase domain to likely serve as an interaction platform for replicase assembly. Further functional interrogation of these determinants led to the identification of one NS3 surface residue on the helicase domain that was essential for virion production without affecting RNA replication. This illustrates that NS3 contains multipurpose protein surfaces allowing the assembly of functionally different protein complexes in a regulated temporal fashion. Taken together, our present data set is providing a basis to further dissect the formation of viral multiprotein complexes required for individual steps of the HCV life cycle.

## Results

### Alanine scanning mutagenesis of surface residues on the NS3 protease domain surrounding L127 identified residues essential for NS5A hyperphosphorylation

**Fig 1:**
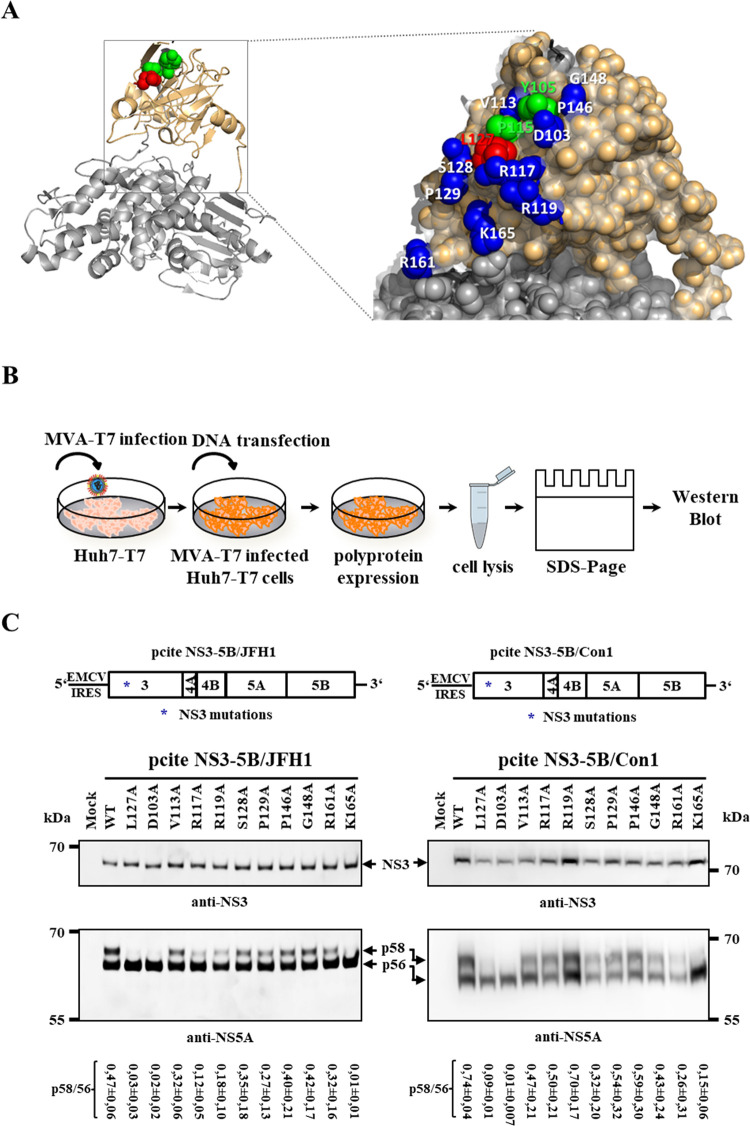
Identification of amino acids on the NS3 protease domain important for NS5A hyperphosporylation. (A) Presentation of the analyzed NS3 protease surface amino acids in the NS3 structure. Left: Ribbon diagram of NS3, highlighting the protease (brown) and helicase (grey) domain. Right: Magnified section of the NS3 protease surface encompassing residue L127 critical for RC assembly. Indicated are surface amino acids (blue spheres) in close proximity to L127 (red sphere) which were analyzed by alanine scanning mutagenesis. Residues (Y105, P115) known from earlier studies [43] to have no effect on NS5A hyperphosphorylation or RNA replication are depicted in green spheres. The illustrations were generated in PyMOL (version 1.10) using coordinates saved in PDB code 1CU1 [57]. (B) Workflow of the MVA-T7^pol^ expression system for replication-independent analysis of polyprotein processing and NS5A hyperphosphorylation of HCV polyprotein derivatives. NS3 mutations were introduced into pcite-NS3-3’ derivatives and plasmids were transfected into Huh7-T7 cells infected with MVA-T7^pol^ vaccinia virus. (C) Top: Schematic representation of the HCV polyprotein expression constructs pcite NS3-5B/JFH1 (left) and pcite NS3-5B/Con1 (right). Polyprotein expression is controlled by an EMCV IRES and encodes the indicated HCV NS3-5B polyprotein of HCV genotype 2a (JFH1, left) or genotype 1b (Con1, right). The positions of NS3 mutations are indicated by an asterisk. Bottom: NS3 mutations were analyzed for their effects on polyprotein processing and NS5A hyperphosphorylation by Western blot. Positions of NS3 and NS5A phospho-isoforms (p56, basal and p58, hyperphosphorylated) are indicated by arrows. Quantification of the p58/p56 ratios based on the Western blots are shown below the respective WB panels in C. The quantifications were performed with ImageJ and are from three experiments (n = 3). The p58/p56 signals were normalized to the respective NS3 signal. Note for this and subsequent figures that the phosphoisoforms of NS5A of isolate JFH-1 have higher apparent molecular weights than 56 and 58 kDa but are still referred to as p56 and p58, respectively.

The molecular events required for the multi-step process of HCV genome replication and virion morphogenesis are only partly understood [30,31]. A central role in this process plays a conserved multifunctional surface area (Y105, P115 and L127; NS3 amino acid numbering) on the NS3 protease domain that is coordinating steps leading to functional HCV replicase assembly by stimulating the NS2^pro^ autoprotease to promote efficient NS2-NS3 cleavage in order to allow NS5A hyperphosphorylation and HCV replicase assembly [43]. Importantly, we found that replacing NS3 L127 by alanine (L127A) interferes with NS5A hyperphosphorylation and functional replicase assembly, thereby blocking viral RNA replication [43]. The importance of NS3 surface interactions for these steps in the HCV life cycle prompted us to search for additional surface residues on the NS3 protease domain surrounding L127 that are required for replicase assembly. Accordingly, we selected phylogenetically conserved surface residues in close proximity to L127 (Fig 1A) that were targeted by alanine scanning mutagenesis. The resulting NS3 mutants were subsequently characterized for their importance in HCV polyprotein processing, NS5A hyperphosphorylation, viral RNA replication and infectious virus production.

In a first step, we tested these mutants for their NS3/NS4A serine protease activity. Accordingly, the mutations were introduced into expression plasmid pcite NS3-NS5B encoding the HCV NS3-NS5B polyprotein to analyze polyprotein processing. Two different genotypes (Con1, genotype 1b and JFH1, genotype 2a) were chosen to distinguish between conserved and genotype-specific effects. We employed the MVA-T7^pol^-mediated expression system in Huh7-T7 cells to monitor the impact of the NS3 mutations on NS3/NS4 protease-mediated HCV polyprotein processing in a replication-independent fashion (Fig 1B; [43]). Western blot analysis revealed that none of the introduced NS3 mutations interfered with authentic NS3-NS5B polyprotein processing in both genotypes as indicated by the production of mature NS3 and NS5A (Fig 1C). Thus, the functionality of NS3/NS4A serine protease complex is not detectably affected by our set of alanine mutations although we cannot exclude subtle kinetic differences of polyprotein cleavage.

In addition, we determined if any of the analyzed surface mutations is interfering with efficient NS2-NS3 cleavage and thus blocking NS3-mediated NS2 protease activation (S1 Fig). Thus, we generated a set of pcite HCV NS2-5B derivatives for genotype 1b and 2a (pcite HCV NS2-5B/Con1 and pcite HCV NS2-5B/JFH1, S1 Fig). We analyzed the effect of the NS3 mutations on NS2-3 cleavage in Huh7-T7 cells by MVA-T7^pol^-mediated expression of HCV NS2-NS5B polyproteins of the two genotypes. Western blot analysis revealed for both genotypes that none of the tested NS3 mutations detectably inhibited NS3-dependent NS2^pro^ activation and NS2-NS3 cleavage (S1B and S1D Fig). Together, these data suggest that the analyzed surface residues have no critical impact on the NS3-mediated NS2^pro^ activation and the functionality of the NS3/NS4A serine protease complex.

Based on our observation that the NS3 L127A mutation is blocking NS5A hyperphosphorylation and viral RNA replication [43], we used the ratio of NS5A phospho-isoforms (e.g., NS5A p56 and p58) as proximation to evaluate if additional NS3 surface residues surrounding L127 are also important for NS5A phosphorylation (Fig 1C; compare NS5A phosphorylation of WT with L127A). When we analyzed NS5A hypo- and hyperphosphorylation after replication-independent expression of the mutated HCV NS3-5B polyproteins, we identified two NS3 mutations (D103A and K165A) that strongly reduced the amount of hyperphosphorylated NS5A isoform(s), similar to the replication-deficient NS3 L127A mutation (Fig 1C). All other analyzed NS3 mutations showed hyperphosphorylated NS5A (Fig 1C). Interestingly, the strong reduction of NS5A hyperphosphorylation caused by D103A and K165A mutations could be observed for both HCV genotypes tested, suggesting a conserved function of these residues. Accordingly, we identified with D103 and K165 two additional NS3 residues on the protease surface which, together with L127, are forming a functional surface required for NS5A hyperphosphorylation.

### The NS3 protease surface residues D103 and K165 constitute, together with L127, a conserved surface area that is important for replicase assembly and viral RNA replication

**Fig 2:**
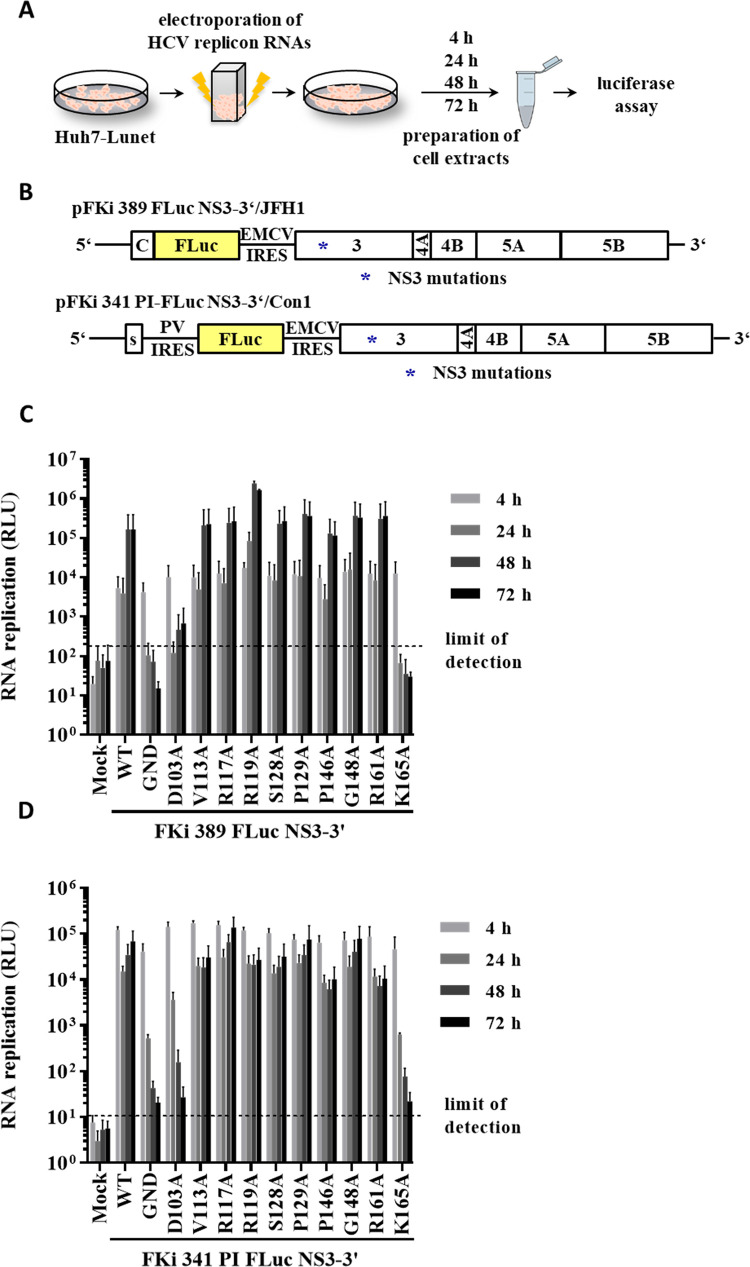
Replication analysis of NS3 protease surface mutations in the JFH1 NS3-3’ replicon identified K165 as critical residue for functional replication complex formation. (A) Workflow to analyze RNA replication ability of HCV replicon derivatives carrying NS3 protease mutations. (B) Schematic representation of the HCV genotype 2a pFKi 389 FLuc NS3-3’/JFH1 replicon (top) and the HCV genotype 1b replicon pFKi 341PI-FLuc NS3-3’/Con1 (bottom). NS3 mutations were engineered into both replicons (indicated by star). (C) Replication analysis of NS3 protease surface mutations in the context of the HCV genotype 2a and (D) genotype 1b. For C and D, the background of the assay is indicated by the horizontal dashed line. *In vitro* transcribed replicon RNAs were electroporated into Huh7 Lunet cells, and luciferase activity was measured at 4, 24, 48 and 72 h post electroporation (pe). Mean values of three independent experiments are shown. Error bars indicate standard deviations.

While the ratio of basal to hyperphosphorylated NS5A can serve as an indicator for RC assembly, the transient replication assay based on reporter replicons allows the determination of functional replication complex assembly (Fig 2A).

Accordingly, all NS3 mutations were introduced into FKi 389 FLuc NS3-3’/JFH1 and FKi 341 PI-Fluc NS3-3’/Con1 replicon constructs to analyze their impact on viral RNA replication (Fig 2B). We used these two replicon systems with different replication characteristics (e.g., the strongly (JFH1) and the weaker (Con1) replicating replicon) to detect conserved and genotype-specific features relevant to HCV RNA replication. *In vitro* transcribed replicon RNAs were electroporated into Huh7 Lunet cells and cells were harvested at the indicated time points to determine *firefly luciferase* levels produced by the respective replicon RNAs (Fig 2B). Wild-type (WT) and replication-deficient GND replicon RNAs served as positive and negative control, respectively. Luciferase values 4 h post electroporation (pe) were used to control for electroporation efficiencies (Fig 2C and 2D). The transient replication assay revealed that at 48 and 72 h pe most NS3 mutants replicated to levels comparable to the respective WT in both genotypes (Fig 2C and 2D). Please note, the marginal increase of luciferase values in the case of the Con1 replicon from 24 h to 72 h pe is a characteristic feature for the widely used replicon derived from the HCV isolate Con1 [58]. As expected from their impact on NS5A hyperphosphorylation, mutations D103A and K165A showed strong negative impacts on viral RNA replication (Fig 2C and 2D). While the K165A mutation blocked RNA replication similar to the replication defective GND control in both genotypes, the D103A mutation abrogated RNA replication when introduced into the more sensitive Con1 replicon but still allowed for low-level RNA replication in context of the more efficient JFH1 replicon system (Fig 2C and 2D). Importantly, our initial analysis of the replicase proteins by the replication-independent MVA-T7^pol^-expression system in Huh7-T7 cells indicated that these inhibitory effects of D103A and K165A on viral RNA replication are not the result of replicase protein translation, protein stability and/or polyprotein processing (Fig 1C).

Together, these experiments led to the identification of two conserved NS3 surface residues (D103 and K165) in close proximity to L127 that were important for NS5A hyperphosphorylation and RNA replication and most likely constitute, together with L127, a functional surface patch on the protease domain required for functional HCV replicase assembly.

### The functional interrogation of helicase domain identified D626 as pivotal determinant for NS5A hyperphosphorylation and viral replicase assembly

**Fig 3:**
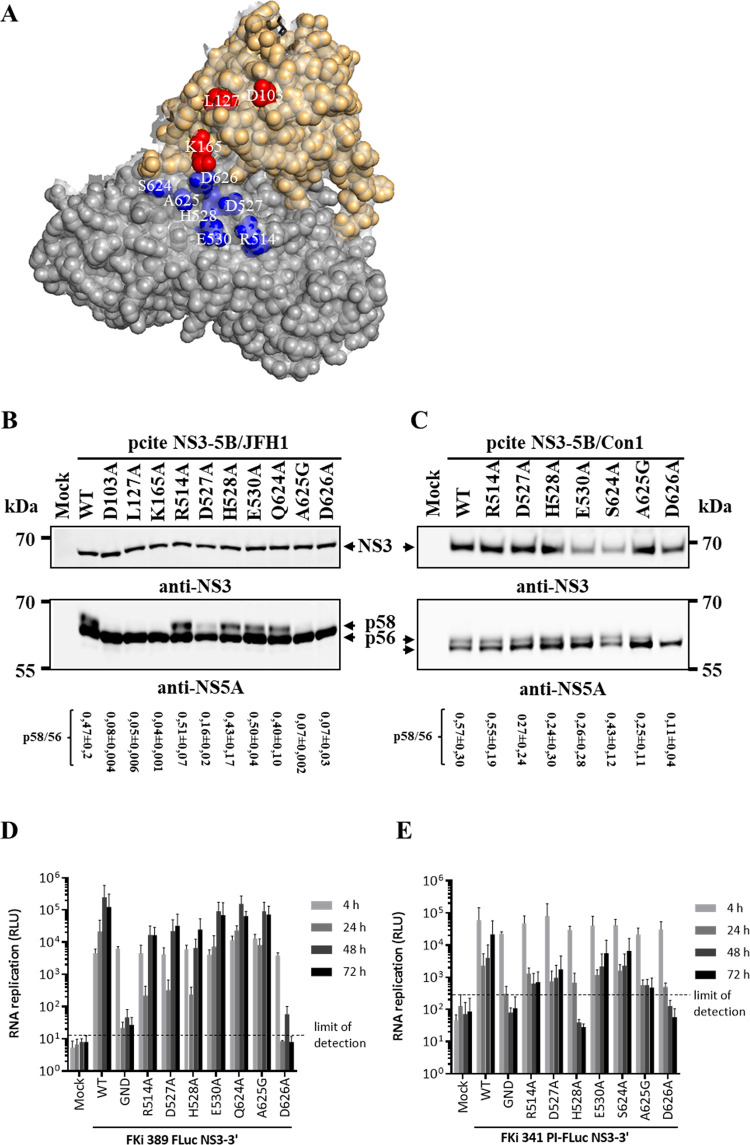
Interrogation of the helicase surface area in close proximity of K165 revealed D626 as important for NS5A hyperphosphorylation and RC assembly. (A) Presentation of the analyzed NS3 helicase surface amino acids. Structural model of NS3 (using coordinates saved in PDB code 1CU1 [57]) showing protease (brown spheres) and helicase (grey spheres) domain surface residues. Red spheres, indicate protease domain residues D103, L127, K165 critical for RC assembly and RNA replication. Blue spheres, indicate surface amino acids of the helicase domain in close proximity to K165 which were analyzed. The illustration was generated in PyMOL (version 1.10). (B and C) MVA-T7^pol^-mediated determination of the effect of selected NS3 helicase mutations on HCV polyprotein processing and NS5A hyperphosphorylation. Indicated NS3 helicase mutations were introduced into pcite NS3-5B/JFH1 (B) and pcite NS3-5B/Con1 (C) expression plasmids derivatives and plasmids were transfected into Huh7-T7 cells infected with MVA-T7^pol^ vaccinia virus. Western blot analysis of HCV polyprotein processing and NS5A hyperphosphorylation of NS3-5B/JFH1 (left) or NS3-5B/Con1 (right) polyprotein derivatives are shown. Positions of NS3 and NS5A phospho-isoforms (p56, basal and p58, hyperphosphorylated) are indicated by arrows. Western blots shown are representative for three independent experiments. Quantification of the p58/p56 ratios based on the Western blots are shown below the WB panels. The p58/p56 signals were normalized to the respective NS3 signal. (D and E) Replication analysis of NS3 helicase mutants in the context of JFH1- (D) or Con1-derived subgenomic replicons (E). Replicon RNAs were introduced into Huh7 Lunet cells by electroporation and luciferase activity (relative light units, RLU) reflecting RNA replication was measured at 4, 24, 48 and 72 h pe. Mean values of three independent experiments are shown. Error bars indicate standard deviations. The background of the replication assay is indicated by the horizontal dashed line. WT: wild type, GND: NS5B replication-deficient mutation.

One important characteristic of the NS3/4A protein complex is the modular structure that is enabling close regulation of the functionally unrelated protease and helicase activities by inter-domain co-operations and allosteric modulation [59–64]. It has been suggested that the NS3/4A complex is oscillating between compact (important for NS3/4A *cis*-cleavage during polyprotein processing) and extended (most likely required for cleavages other than NS3-4A occurring *in cis* during polyprotein processing and RNA unwinding) conformations to serve its multiple functions during the viral life cycle [12,57,61,63,65,66]. Interestingly, in the compact conformation the important K165 residue is in close proximity to helicase surface residues (Fig 3A, [57]). Based on this observation we were wondering whether the multifunctional surface patch important for replicase assembly extends into the NS3 helicase domain.

Accordingly, we used the available closed conformation structure of NS3 to select helicase surface residues R514, D527, H528, H530, Q624 (S624 in genotype 1b), A625, D626 in close proximity to K165 in this conformation that were replace individually by alanine residues (Fig 3A). These mutations were analyzed in the context of genotype 1b (Con1) and genotype 2a (JFH1) for their impact on NS5A phosphorylation and RNA replication, respectively. MVA-T7^pol^-assisted replication-independent NS3-5B polyprotein expression in Huh7-T7 cells followed by Western blot analysis revealed that polyprotein processing was unaffected for all tested NS3 helicase mutations indicating that none of these residues is detectably interfering with the NS3/4A protease activity (Fig 3B).

However, differences concerning the NS5A phosphorylation were detected for some NS3 helicase mutants when compared to WT. In the NS3-5B/JFH1 genotype 2a context most of the helicase mutations (R514A, H528A, E530A, Q624A) did allow for WT-like or reduced (D527A) levels of NS5A hyperphosphorylation (Fig 3B). In the NS3-5B/Con1 polyprotein we also observed comparable NS5A phosphorylation ratios for most helicase mutants (Fig 3C). Interestingly, a genotype-specific difference for NS5A hyperphosphorylation was detected for mutation A625G: in the NS3-5B/JFH1 polyprotein this mutation strongly interfered with NS5A hyperphosphorylation (Fig 3B), while the same mutation still permitted reduced but detectable NS5A hyperphosphorylation in the NS3-5B/Con1 context (Fig 3C). Importantly, the D626A mutation, which is adjacent to K165 in the compact NS3 structure (Fig 3A, [57]), inhibited detectable NS5A hyperphosphorylation in both genotypes (Fig 3B and 3C) suggesting that D626 represents another important determinant for the assembly of a functional replicase, similar to surface residues D103, L127 and K165.

To test this assumption, the helicase mutations were introduced into the pFKi-389-Luc/NS3-3’_JFH1 and the pFKi-341-Luc/NS3-3’_Con1 replicon plasmids. In the efficiently replicating JFH1 reporter replicon system most of the tested helicase mutations supported RNA replication, albeit with different efficiencies: mutants E530A, Q624A and A625G replicated with WT-like efficiencies while mutants R514, D527A and H528A showed a delayed and reduced RNA replication phenotype (Fig 3D). Interestingly, and in agreement with the observed D626A-mediated inhibition of NS5A hyperphosphorylation, the D626A mutation blocked viral RNA replication (Fig 3D). The introduction of the NS3 helicase mutations into the less efficient genotype 1b replicon system revealed some genotype-specific differences regarding their impact on viral RNA replication. The replicon mutants E530A and S624A replicated at slightly reduced levels while the other helicase mutations either led to a strong reduction (R514, D527A and A625G) or in the case of H528A to an inhibition of RNA replication (Fig 3E). Interestingly, in the JFH1 genotype 2a the A625G mutation strongly reduced NS5A hyperphosphorylation but allowed for efficient RNA replication while in the Con1/1b genotype the same mutation led to a strongly reduced but detectable NS5A hyperphosphorylation and a strong reduction in RNA replication (compare A625G in Fig 3B–3E). The ability of A625G to replicate was unexpected because of the strong correlation between NS5A hyperphosphorylation and RNA replication throughout this study. Importantly, the D626A mutation also inhibited viral RNA replication of the genotype 1b replicon. Thus, D626 represents another conserved critical determinant for functional replicase assembly. Together, these data reveal that D626 on the helicase domain constitutes, together with D103, L127 and K165 on the protease domain, a multifunctional surface area that appears to functionally connect the NS3 protease and helicase domains to support replicase assembly.

### Permutation analysis of the NS3 surface residues D103, L127, K165 and D626 reveals different requirements for these conserved amino acids to support replicase assembly

**Fig 4:**
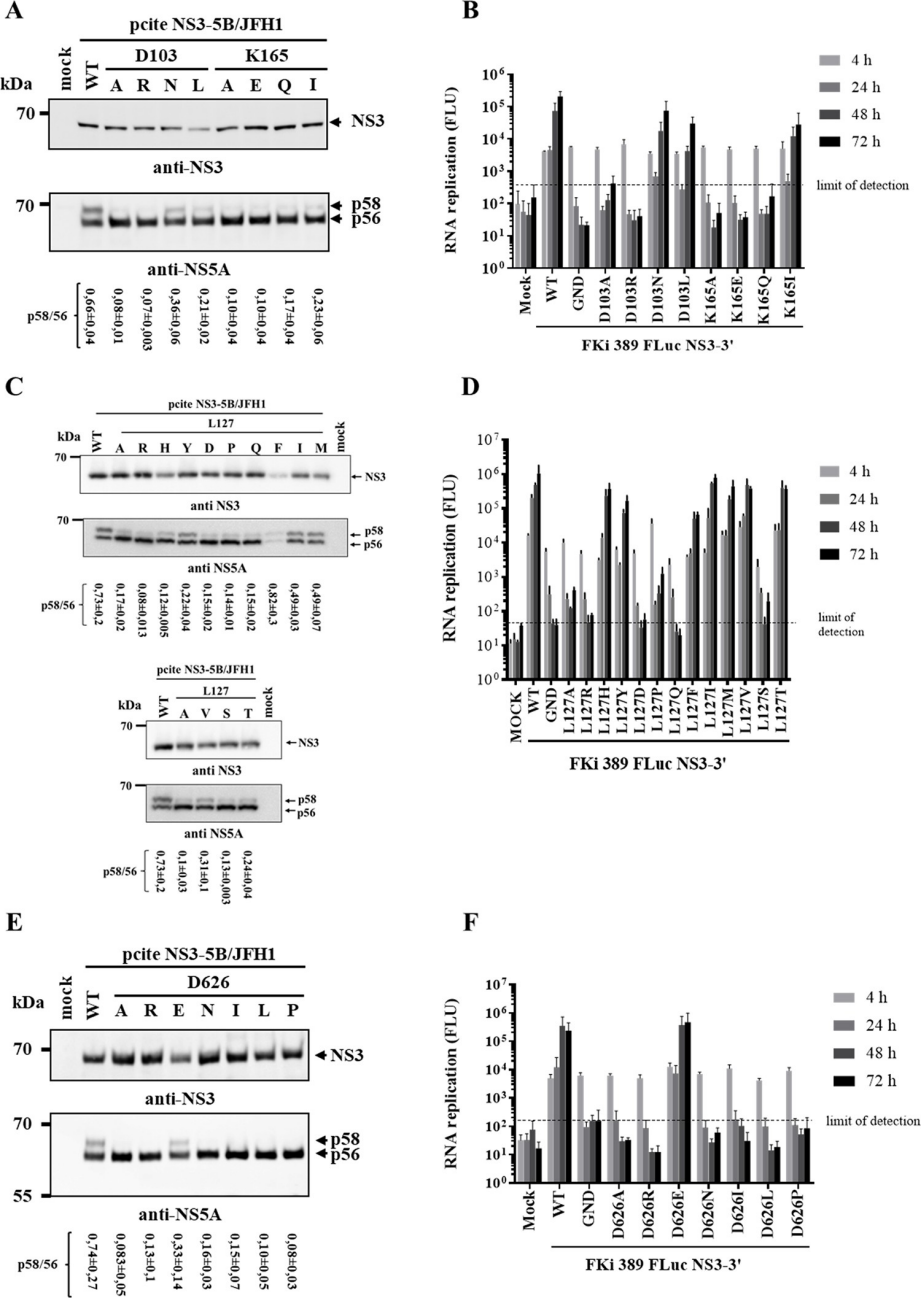
Characterization of amino acid requirements at critical NS3 surface positions by limited permutation analysis of D103, L127, K165 and D626. NS3 permutations mutations were introduced into pcite NS3-5B/JFH1 to analyze effects of the NS3 permutations on NS5A hyperphosphorylation in the MVA-T7-^pol^ expression system in Huh7-T7 cells. NS3 permutations in pFKi 389 FLuc NS3-3’/JFH1 were applied to determine their impact on viral RNA replication in Huh7 Lunet cells. (A, C and E) Analysis of HCV polyprotein processing and NS5A hyperphosphorylation by anti-NS3 and NS5A-specific Western blot. Western blots shown are representative for three independent experiments. Quantification of the p58/p56 ratios based on the Western blots are shown below the respective WB panels. The quantifications were performed with ImageJ and are from three experiments (n = 3). The p58/p56 signals were normalized to the respective NS3 signal. (B, D and F) RNA replication analysis of D103, K165 and D626 permutations. RNA replication capacities of mutated replicon RNAs in Huh7 Lunet cells was measured by determining *firefly* luciferase activities (FLU) at 4, 24, 48 and 72 h pe. Mean values of three independent experiments are shown. Error bars indicate standard deviations. The background of the replication assay is indicated by the horizontal dashed line. WT: wild type, GND: NS5B replication-deficient mutation.

The conservation of the NS3 residues critical for replicase assembly among different HCV genotypes emphasizes their functional importance but does not provide insights into which amino acid characteristics are required at those positions to support NS5A hyperphosphorylation and viral RNA replication. However, an interesting aspect is the different amino acid character (charged for D103, K165 and D626; hydrophobic for L127) of those amino acids. Therefore, we conducted a permutation analysis to elucidate the amino acid character required at the individual critical NS3 positions (D103, L127 K165 and D626). Accordingly, we introduced either charge-flip (D103R, K165E, D626R), polar (D103N, K165Q, D626N) or hydrophobic (D103L, K165I, D626I, D626L and D626P) amino acid exchanges at positions with charged residues. For the L127 residue we could recently show that a hydrophobic exchange (L127F) at this position did not interfere with L127’s functionality during the NS3-mediated NS2^pro^ activation while a charged residue (L127R) at this position strongly reduced the NS3-mediated NS2^pro^ activation suggesting that hydrophobic protein-protein interactions are required for this function [43]. However, those mutations were not analyzed for their impact on NS5A hyperphosphorylation and replicase assembly. Therefore, we included those mutations in our permutation analysis and also changed L127 to charged (L127D), hydrophobic (L127M, V, P and I) and polar (Q Y, H, S) residues. All permutations were analyzed for polyprotein processing and NS5A hyperphosphorylation in the NS3-5B/JFH1 polyprotein and for RNA replication in the pFKi 389 FLuc NS3-3’/JFH1 replicon system (Fig 4).

Replication-independent polyprotein expression in the MVA-T7^pol^ system in Huh7-T7 cells demonstrated that all analyzed NS3 permutations allowed for functional polyprotein processing further confirming that these residues do not play important roles for the NS3/4A serine protease functionality (Fig 4A, 4C and 4E). The analysis of permutations of the charged residues D103 and K165 revealed that charge flip permutations at both positions (D103R, K165E) strongly reduced NS5A hyperphosphorylation (Fig 4A) and blocked RNA replication (Fig 4B). Substitutions with a hydrophobic residue (D103L, K165I) or with polar residues (D103N, K165Q) allowed for reduced but detectable level of NS5A hyperphosphorylation compared to WT (Fig 4A). Compared to D103 permutations, K165 permutations seemed to affect NS5A hyperphosphorylation more strongly (Fig 4A). Interestingly, changes to hydrophobic amino acids (D103L, K165I) as well as the polar D103N permutation supported viral RNA replication (Fig 4B). In contrast, a replicon RNA carrying the polar K165Q exchange only replicated at very low levels (Fig 4B). The permutation analysis of L127 showed broader flexibility. Different hydrophobic (L127V, L127M, L127I and L127F), one potentially charged (L127H) and two polar (L127T and L127Y) amino acids were tolerated with respect to NS5A hyperphosphorylation and RNA replication, however, to different degrees (Fig 4C and 4D).

In contrast, the presence of charged residues L127R and L127D as well as the polar exchanges L127Q and L127S were found to strongly reduce or inhibit those functions (Fig 4C and 4D).

In the case of the acidic D626 residue, we found that only the conservative D626E exchange was functional with regard to NS5A hyperphosphorylation and RNA replication, while charge-flip (D626R), hydrophobic (D626I, P and L) and polar (D626N) exchanges failed to support these processes (Fig 4E and 4F). Together these experiments reveal different requirements for the critical surface residues on the protease and helicase domain: residues on protease domain surface (D103, L127 and K165) exhibit some flexibility with regard to amino acid character to retain their functionality. In contrast, the critical helicase residue D626 requires an acidic amino acid character to allow functional replicase assembly to occur.

### Identification of helicase surface residues critical for the production of infectious virus

**Fig 5:**
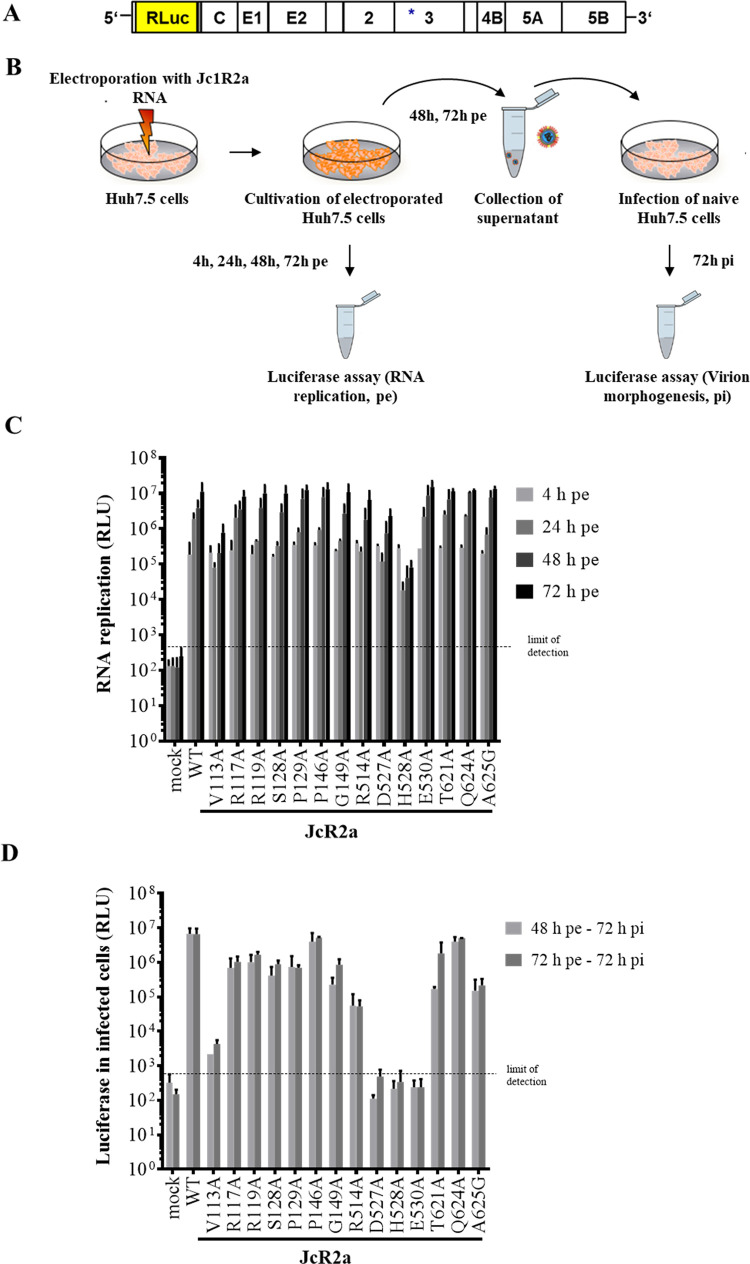
Impact of replication-competent NS3 surface mutations on the HCV genome replication and infectivity release. (A) Schematic diagram of the full-length monocistronic reporter virus (JcR2a; [70]). The HCV proteins are represented as white boxes, *Renilla* luciferase (RLuc) used for indirect quantitative analysis of HCV genome is indicated by a yellow box and the NTRs are represented by black lines, respectively. The positions of NS3 aa substitutions are indicated by a star. (B) Scheme of the experimental design. (C) Determination of viral genome replication. Huh7.5 cells were electroporated with *in vitro* transcribed viral RNA and kinetics of HCV genome replication were quantified 4, 24, 48 and 72 hours post electroporation by measuring *Renilla* luciferase activity (relative light units, RLU). (D) Analysis of virion morphogenesis. Supernatants containing released infectious particles were harvested 48 and 72 hours post electroporation and used for inoculation of naïve Huh7.5 cells. Three days after infection, cells were harvested, cell lysates were prepared, and intracellular luciferase activity was determined. In panels (C) and (D) representative results of three independent experiments with standard deviations are shown. The background of the luciferase assay is indicated by the horizontal dashed line.

The NS3/NS4A complex not only functions in polyprotein processing and RNA replication but also during HCV virion morphogenesis and residues critical for HCV assembly have been mapped to different regions of the NS3 protein, including the helicase domain, the N-terminal α0 helix of the protease or the linker region connecting protease and helicase domain [33,44,67–69]. To investigate whether the multifunctional NS3 surface area identified in this study also contributes to virion morphogenesis, we engineered the NS3 surface mutations into the monocistronic *Renilla* luciferase reporter virus (JcR2a) that is based on the intragenotypic genotype 2a chimera Jc1 (Fig 5A; [70]).

The *in vitro* transcribed full-length JcR2a RNAs representing wild type (WT), the respective NS3 mutants, or the NS5B polymerase defective GND mutant were electroporated into Huh7.5 cells and incubated for the indicated times. *Renilla* luciferase activities were measured in cell lysates to determine viral genome replication while the supernatants from the electroporated cells were used to infect naïve Huh7.5 cells to analyze infectious virus production (Fig 5B).

Luciferase activities measured in cell lysates of electroporated cells collected at 4 h pe confirmed comparable transfection efficiencies for the different JcR2a RNAs (Figs 5C and S2A). Their capacity for viral genome replication was determined at 24, 48 and 72 h pe, respectively (Fig 5C and S2A). In agreement with our observations in the replicon system, all replication-competent NS3 mutants introduced into full-length JcR2a replicated, albeit at different levels (Fig 5C). While most mutants showed genome replication comparable to WT, NS3 mutants V113A and D527A produced lower luciferase levels (approx. 10-fold) and especially H528A exhibited a strongly reduced (approx. 100-fold) RNA replication phenotype (Fig 5C). Also in agreement with the replicon data, NS3 mutants D103A, K165A and D626A did not replicate in the full-length JcR2a context (S2A Fig).

Next, we determined the influence of these NS3 surface mutations on infectious virus production by inoculating naïve Huh7.5 cells with supernatants from electroporated cells collected at 48 or 72 h pe (Fig 5B). In this experimental setting, the values of luciferase activity produced from the infected cells represented a measure of virus production by the electroporated cells. As shown in Fig 5D, only JcR2a mutants P146A and Q624A produced amounts of infectious virus comparable to WT, while NS3 mutations R117A, R119A, S128A, P129A, R514A, T621A and A625G produced lower (approx. 5-10-fold) amounts of infectious virus which in most cases reflects the slightly lower replication capacity of these mutants (Fig 5D). Mutations V113A, D527A, H528A and E530A exhibited either strongly reduced levels (V113A; >3 log_10_) or a nearly complete block (D527A, H528A and E530A) of infectious virus production (Fig 5D), while the NS3 mutants D103A, K165A and D626A did not support virion morphogenesis as expected due to their severe impact on RNA replication (S2B Fig). The differences in virion morphogenesis detected by measuring *Renilla* luciferase levels in infected cells were confirmed when we determined virus titers (50% tissue culture infective dose per ml (TCID_50_/ml)) for the selected JcR2a mutants R514A, D527A, H528A, E530A and A625G (S2C Fig). The strongly reduced or inhibited virus production for the JcR2a derivatives V113A and H528A could in part be explained by their lower replication phenotype in the full-length genome context (compare Fig 5C and 5D). In contrast, the mutations D527A and especially E530A supported viral genome replication at either moderately (D527A) or slightly reduced (E530A) levels compared to WT (Fig 5C) but were severely inhibited in their capacity to produce progeny virus (Fig 5D), indicating that these residues are selectively important for virion morphogenesis. Together with our observation that D626 is important for functional replicase assembly and viral RNA replication these results reveal that HCV uses a multifunctional NS3 helicase surface area surrounding residues D527, H528 and E530 to coordinate protein interactions important for virion morphogenesis.

### The helicase surface mutations D527A and E530A block virus assembly

**Fig 6:**
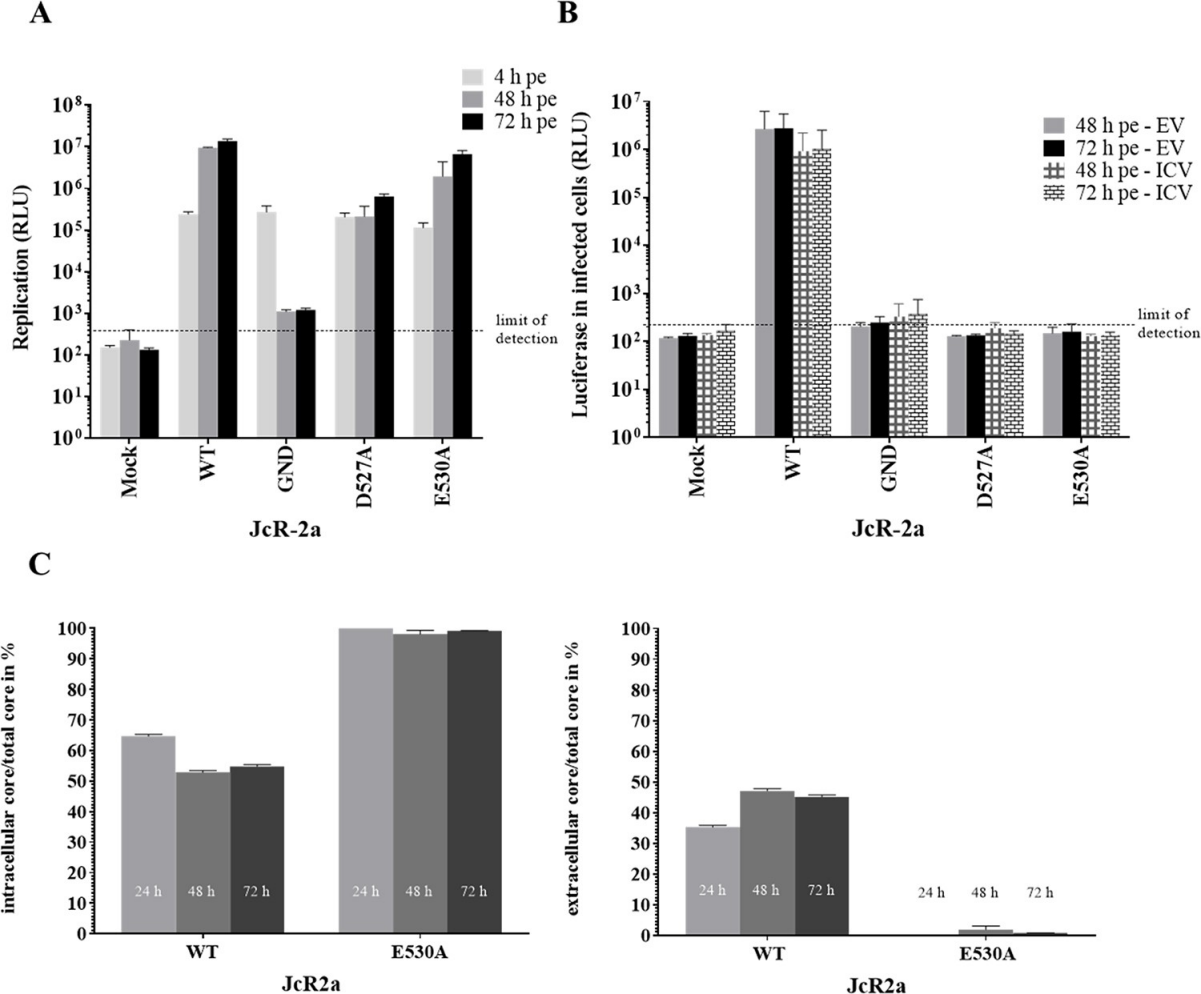
NS3 mutations D527A and E530A inhibit viral particle production prior to virion release and before the formation of infectious intracellular virions. (A, B) The NS3 surface mutations D527A and E530A inhibit the formation of infectious intra- and extracellular virus particles. (A) Huh7.5 cells were electroporated with HCV JcR2a RNA genomes specified at the bottom of the graph and 4, 48 and 72 h later, their replication capacities were determined by *Renilla* luciferase assay using cell lysates. (B) The cell-associated (ICV) and extracellular (EV) infectivity was determined by inoculating naïve Huh7.5 cells with cell lysates or culture supernatants of transfected cells and measuring *Renilla* luciferase activity 72 h pi. For each time point the amounts of ICV and EV infectivity are given. Mean and SEM of three independent experiments are shown. (A, B) The background of the assay is indicated by the horizontal dashed line. (C) The E530A mutation causes a massive block in core protein release. To determine amounts of intra- (left panel) and extracellular (right panel) core protein, cell lysates and cell culture supernatant from Huh7.5 cells electroporated with WT or E530A HCV JcR2a RNA genomes were subjected chemiluminescent microparticle immunoassay (CMIA), respectively. The intracellular (left panel) and the released (extracellular, right panel) core protein amount was normalized to total core protein amount (intra- and extracellular).

The specific defect in infectious virion production observed for mutants D527A and E530A may either be caused by the inhibition of virus assembly, the block of infectious virus particle release or an interference with nucleocapsid formation. To determine whether mutants D527A and E530A are still able to promote virus assembly but are defective in the release of infectious virus particles, we individually determined their cell-associated (ICV) and the cell-released (EV) infectivity. Accordingly, Huh7.5 cells were electroporated with WT JcR2a RNA or mutants D527A or E530A. Replication was monitored at indicated time points pe and confirmed comparable replication capacities between WT and E530A as well as a slightly lower replication level for mutant D527A (Fig 6A).

We used supernatants or lysates generated by freeze-thaw cycles from these electroporated cells to infect naïve Huh7.5 cells. As shown in Fig 6B, we detected cell-associated (ICV) and cell-released (EV) infectivity for the WT virus. Furthermore, we observed that JcR2a mutants D527A and E530A did not support the production of infectious extracellular virus particles (Fig 6B). Importantly, we found that these mutations also blocked the generation of cell-associated, intracellular infectivity (Fig 6B) arguing for a specific impairment of HCV assembly rather than virion release. Taken together, these results indicate that the NS3 mutations D527A and E530A impair virus particle production prior to the formation of fully infectious virions.

These observations indicate that mutating these NS3 surface residues is either resulting in the release of non-infectious, core-containing particles or in the block of viral nucleocapsid envelopment. To discriminate between these possibilities, we next measured the release of core protein by a commercial chemiluminescent microparticle immunoassay (CMIA) to test for release of non-infectious core-containing particles by these mutant viral genomes. We performed this analysis with WT JcR2a and the NS3 mutant E530A because JcR2a E530A showed only a slightly lower replication capacity compared to WT (Fig 6A). Accordingly, we electroporated Huh7.5 cells with either WT or E530A JcR2a viral RNAs and quantified the amount of intracellular or extracellular core protein relative to total core protein by CMIA (Fig 6C). At 72 h pe, we detected approx. 3 fold less total core amount for the E530A variant compared to WT, consistent with the slightly lower replication capacity of this NS3 mutant (Fig 6A). Importantly, only very low amounts of core protein were released from cells transfected with the E530A mutant, in stark contrast to JcR2a WT for which up to 50% of total core was detectable in the culture supernatant (Fig 6C, compare left and right panel). This result argues for a strong defect of the E530A mutant in virus assembly (Fig 6C). Together, these observations suggest that the NS3 surface mutation E530A (and most likely also D527A) is interfering with an early step of HCV particle assembly as there is no infectivity in cells and also no core release. One possible scenario could be that non-functional capsids are formed.

#### The E530A mutation is not interfering with viral nucleocapsid envelopment

**Fig 7:**
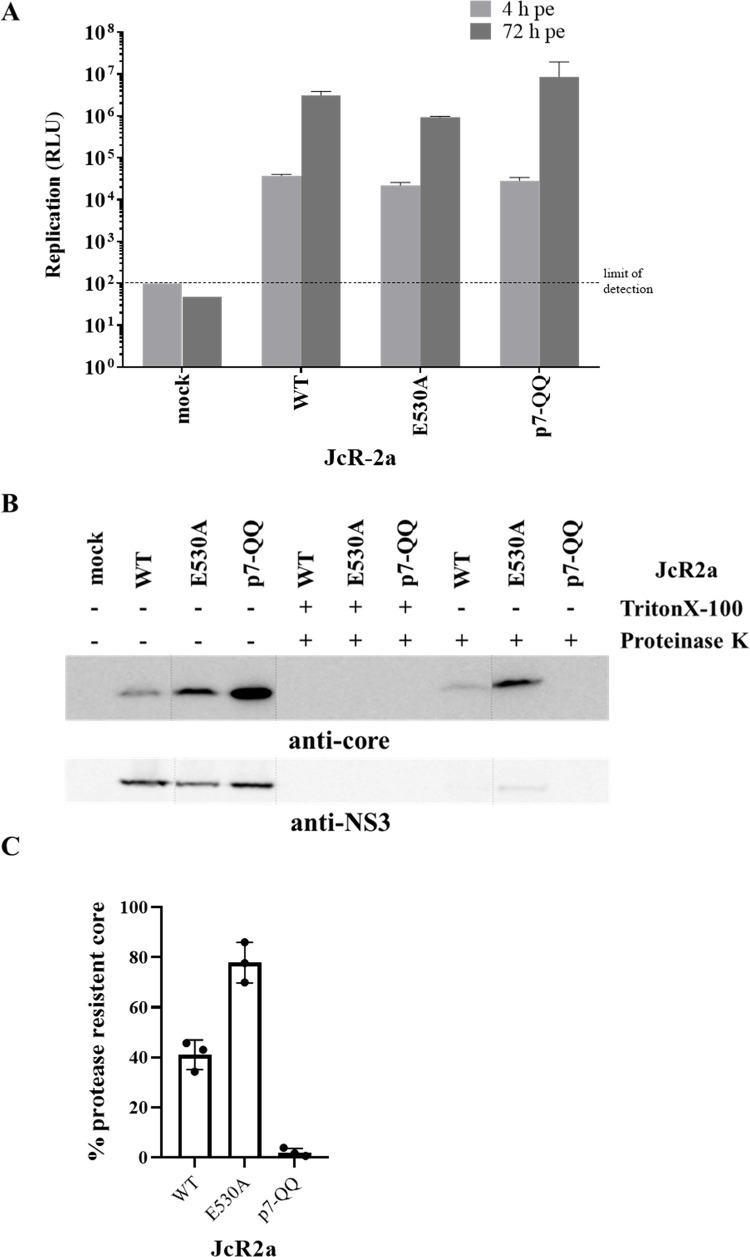
NS3 mutation E530A do not interfere with HCV nucleocapsid envelopment. (A) Huh7.5 cells were electroporated with indicated HCV RNAs and viral RNA replication was analyzed at 4 h and 72 h pe. The background of the replication assay is indicated by the horizontal dashed line. (B) Cell lysates (72 h pe) were prepared and either mock treated or incubated with 50 μg/ml proteinase K for 40 min on ice. As positive control, samples were treated with 1% Triton X-100 prior to proteinase K digestion. Core and NS3 protein detection by core- (C7/50) and NS3-specific (3E2) Western blot is shown. Shown is a representative Western blot of three independent experiments. (C) The density of bands corresponding to core in each treatment was scanned and quantified. The percentages of core levels detected in samples treated with proteinase K alone relative to that of an untreated sample were determined. The diagram represents the mean of the 3 experiments with standard deviation and the variation shown.

The observed block of virion assembly can be caused by inhibition of distinct steps such as core trafficking to the lipid droplets (LDs), core oligomerization, nucleocapsid formation, or core envelopment. To assess whether the envelopment of HCV nucleocapsids is affected by the NS3 mutations, we determined resistance of intracellular core protein against treatment with proteinase K (PK). Because the viral RNA in infectious HCV virions is protected by the core protein and the surrounding lipid envelope [71–73], it should be resistant against PK degradation [74]. Using this assay, we determined if the E530A mutation prevented viral envelopment, thus rendering HCV core protein sensitive to degradation by PK. As positive controls, samples were treated with TritonX-100 to remove the lipid envelope and we included a JcR2a mutant carrying two point mutations in p7 (p7-KR33/35QQ) known to disrupt virion envelopment [74,75]. Huh7.5 cells electroporated with genomic *in vitro* transcripts were harvested 72 h pe and cell lysates were prepared by freeze-thaw cycles and analyzed for RNA replication by luciferase assay and core protein sensitivity to PK by using Western blot. For each sample we confirmed viral replication at 72 h pe (Fig 7A).

Moreover, in the absence of PK treatment, core and NS3 were readily detected in lysates of transfected cells although amounts of NS3 were somewhat lower in case of the NS3 mutant compared to WT and the p7 mutant most likely reflecting a slightly lower replication of the JcR2a E530A derivative relative to WT and the p7 mutant (Fig 7A and 7B). As expected, in samples treated with Triton-X100, core and NS3 were degraded by PK treatment. In the absence of detergent, core protein was resistant to PK to deferent degrees in the case of the WT and the E530A NS3 mutant, whereas it was undetectable with the p7 mutant (Fig 7B). Quantification of the Western blot revealed ~40% and 80% of core protein being resistant to PK in case of the WT and the E530A NS3 mutant, respectively (Fig 7C). Taken together, these results suggest that the E530A mutation does not detectably interfere with core envelopment. One possible explanation for the observed PK resistance of core protein in the case of the E530A mutant could be the formation of aberrant non-functional core structures. Of note, such non-functional core structures could also explain its lack of infectivity. To address this aspect, we separated core protein complexes in absence of detergent in continuous 0–30% sucrose density gradients. While there was a single prominent peak of rapidly sedimenting core in fraction 9 for WT JcR2a (S3 Fig), the E530A mutant showed a behavior similar to the packaging-defective JcR2a-p7QQ [74] by sedimenting more slowly in fraction 6 (S3 Fig). The results of these sedimentation assays strongly argue for aberrant core aggregates. Together, the PK assay and the core sedimentation assay suggest a defect in proper nucleocapsid formation for JcR2a E530A that could explain the lack of core release and the lack of infectivity observed for this mutant.

## Discussion

All plus-strand RNA viruses share common characteristics of genome replication, including an RNA-dependent RNA polymerase, the need to regulate genome translation versus genome replication, the compartmentalization of replicase complexes within membranous compartments as well as the recruitment of viral proteins to putative assembly sites, where virus particles are formed. In the case of HCV, viral RNA replication requires NS3, NS4A, NS4B, NS5A, and NS5B together with an unknown set of cellular proteins. However, we do not yet completely understand how these proteins work and interact with each other to function as a macromolecular machine. Given that HCV has become an important model system for positive-strand RNA virus replication, understanding the fundamental mechanisms of its replication will likely inform us also about how other viruses of this type do replicate.

**Fig 8:**
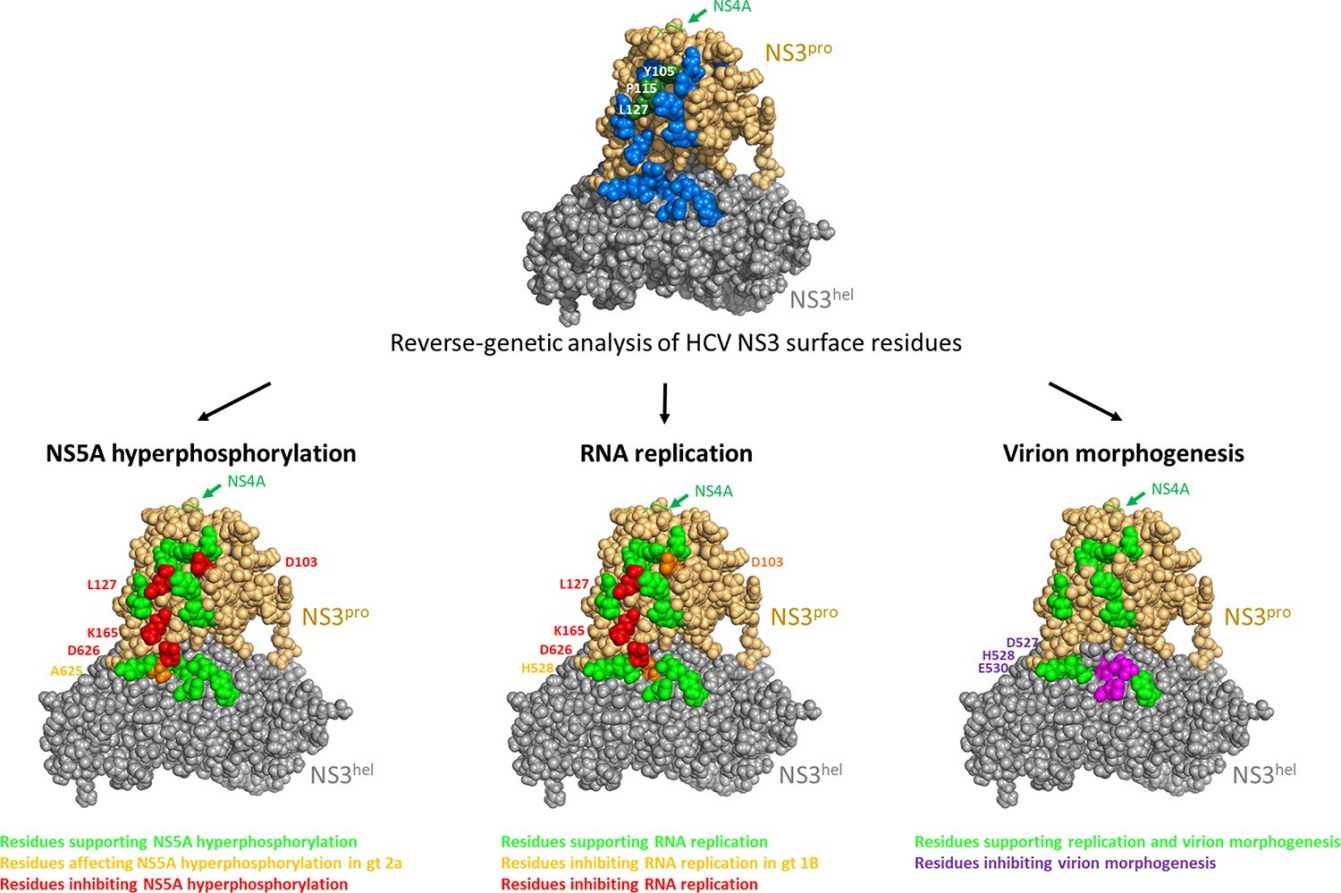
Summary of the reverse-genetics characterization of a multipurpose NS3 Surface patch coordinating HCV replicase assembly and virion morphogenesis. NS3 surface residues important for NS5A hyperphosphorylation, RNA replication or virion morphogenesis are highlighted in red or purple. The exit position of the NS4A cofactor is indicated by a green arrow.

Our results from this study demonstrate that the multifunctional NS3 protein uses a defined surface area on the protease and helicase domain to undergo a step-wise series of protein-protein interactions required for different steps of the HCV life cycle. We observed that D103 and K165, together with L127, constitute a functional platform on the NS3 protease surface that is required for NS5A hyperphosphorylation (Fig 1) and RNA replication (Fig 2). These results are extending earlier findings that NS5A phosphorylation is a process that is regulated by a complex interplay between various viral and cellular proteins within the viral replicase and is critical for RNA replication [21,25,43,76,77]. Recently it was demonstrated that sequential NS5A phosphorylation requires the ATP-binding domain of NS3 helicase domain [77]. Thus, identifying surface mutations within the protease domain, which are also important for NS5A hyperphosphorylation suggests that NS3 might modulate NS5A phosphorylation status by inter-domain co-operations between NS3 protease and helicase domains. The observation that the two residues in the protease domain (D103 and K165) are important for NS5A hyperphosphorylation and RNA replication in two different HCV genotypes points to the general significance of our findings. Interestingly, while both residues, when replaced by alanine, block NS5A hyperphosphorylation (Fig 1), the mutants differ slightly in RNA replication: While mutation D103A blocks RNA replication in a genotype 1b replicon it allows low-level replication in the more efficient genotype 2a isolate JFH-1. The exchange of K165 to alanine impairs RNA replication in both replicon systems, similar to our observations for the L127A mutation ([43]; Figs 2 and 8).

One possible explanation for these differences could be that both residues (D103 and K165) are contributing to the assembly of the viral replicase *via* different protein interactions. In a current model of the NS3/4A complex, the C-terminal 19 amino acid of NS4A form a so-called kink and acidic region protruding from the NS3 protease domain [57,78]. Because the D103 surface residue is in close proximity to the NS4A protruding site, D103 could modulate interactions involving the C-terminal portion of NS4A (Fig 8). This acidic region has been proposed to dynamically regulate interaction of NS3/4A with other components of the viral replicase, leading to formation of NS3/4A-containing multi-protein complexes promoting NS5A hyperphosphorylation and replicase assembly [78].

In contrast, K165 is located in close proximity to the protease-helicase domain interface that has been suggested to regulate conformational changes between an open and a closed conformation in the full-length NS3 protein [59–61,63,79,80]. Interestingly, our extended analysis of selected NS3 helicase domain surface residues revealed that D626 is also required for NS5A hyperphosphorylation and RNA replication in both HCV genotypes, similar to K165. In contrast, mutation H528A blocks RNA replication only in a genotype 1b replicon but allows replication in the more efficient genotype 2a replicon, again reflecting genotype-specific differences similar to the D103A mutation (Figs 3 and 8). Similar to H528A, a genotype-specific difference was also detected for the A625G mutation. While replicating similar to WT in the JFH1 replicon, the same mutation strongly attenuated the Con1 replicon system (Fig 3). This observation was somewhat unexpected since A625G mutation blocks detectable NS5A hyperphosphorylation in the JFH1 context to a similar degree than the non-replicating K165A or D626A mutations (Fig 3B). Interestingly, a similar lack of correlation between the NS5A phosphorylation and replication has been described for the NS5A mutation S225A [25,81]. In a possible scenario the NS3 A625G mutation might change the interaction between NS5A and PI4KIIIα, leading to enhanced levels of p56 [82]. However, our understanding of the complexity of NS5A phosphorylation is still incomplete due to the multiple phosphorylation events taking place and further work is required to fully characterize both the kinases involved and the consequences of different NS5A phosphorylation in the virus life cycle.

The reasons for these genotype-specific differences are currently unknown. Different effects of the respective alanine mutation on the local protein fold and/or differences in local surface interactions might account for these observed genotype-specific differences. H528 is involved in interactions with the NS3 C terminus by interacting with V629 thereby positioning the NS3 C terminus in proximity to the protease active site. Most of the amino acids accommodating this interaction are conserved between genotypes. However, adjacent to V629 genotype-specific differences can be found at position 630 (V630/gt1b; M630/gt2a). It is possible that the larger sidechain of M630 better supports NS3 conformations required for RNA replication compared to V630. D626 is part of C-terminal helicase residues, which bind in the active site of the protease domain and undergo backbone hydrogen-bonding interactions, resulting in the formation of an antiparallel β-sheet [57]. These interactions are proposed to stabilize NS3 in a closed conformation as the result of *cis*-cleavage between NS3 and NS4A. While the compact NS3 conformation is enabling NS3-NS4A *cis*-cleavage during polyprotein processing, an extended NS3 conformation in complex with NS4A is most likely required for downstream polyprotein processing *in trans* and its nucleic acids unwinding activity [12,61,81]. To undergo such a conformational change NS3 would need a step that disrupts the interaction between the C-terminal ß-strand and the protease domain [61]. Due to the innate affinity between the NS3 C-terminal ß-strand and the protease domain, it was speculated NS3 might alternate between compact and extended conformations even during RNA binding/unwinding and/or packaging of the RNA genome into infectious viral particles.

The modular domain structure of NS3 is enabling close regulation of the protease and helicase activities not only by inter-domain co-operations, but also by allosteric modulation [79,80,82]. Accordingly, mutating K165 and D626 could interfere with these inter-domain co-operations. Interestingly, in the structure of the closed conformation of the NS3/NS4A complex, the acidic sidechain of D626 forms a weak, solvent-exposed salt linkage with the amino group of K165 [57]. Although it is unclear whether this interaction is important for stability of the closed conformation, our finding that both residues are pivotal for NS5A hyperphosphorylation and RNA replication suggests that these conserved residues serve multiple functions (Figs 2 and 3). Because these conformational changes are considered to be pivotal for HCV replicase formation [62,82], compromising these co-operations by mutating K165 or D626 would interfere with the ability of the NS3/4A protein complex to adopt optimal conformation(s) required for RNA replication. Alternatively, HCV replicase assembly could rely on interdependent sets of NS3/NS4A-containing protein complexes and composite polyprotein complex formation to support viral replication [83] or these surface residues could interact with cellular proteins that are functional parts of the active HCV replicase complex. The related dengue virus NS3 protein also has been reported to adopt an equilibrium between alternative extended and compact conformations, highlighting the biological importance of NS3 inter-domain interactions and allosteric modulation across the *Flaviviridae* family [84].

Besides identifying a patch of NS3 surface residues important for NS5A hyperphosphorylation and replicase assembly, we also found that adjacent to the critical D626 residue in the helicase domain a small cluster of surface residues (D527, H528 and E530) is important for virion morphogenesis (Figs 5 and 8). While D527A and H528A mutations also reduced viral RNA replication, the E530 surface residue is pivotal for virus particle formation without affecting viral genome replication (Fig 5 and 8). The NS3 E530A mutation blocks an early step in HCV virion morphogenesis since we could not detect infectious intracellular particles, although envelopment of nucleocapsids was not compromised (Fig 7). The underlying mechanism how this mutation impairs formation of infectious virions is still unknown. In NS2 pull-down experiments we did not observe differences with regard to the amounts of NS3, core and NS5A that were co-precipitated when comparing wild-type and the E530A mutant (S4 Fig). However, we note that these interactions might be indirect and take place at the DRMs (detergent resistant membranes—lipid-raft-like structures), which were shown to be sites where E1/E2 glycoproteins, NS2, NS3, NS5A, and core accumulate [19,85–89]. Furthermore, we did not detect differences with regard to the formation of E2-NS5A double-positive structures in close proximity to lipid droplets when comparing WT NS3 with the packaging-defective E530A variant (S5 Fig). Thus, the assembly-dependent subcellular relocalization of E2 is still possible when employing a replication-competent sub-genomic (NS3-NS5B)NS5A-mCherry replicon carrying the NS3-E530A mutation (S5 Fig). This observation suggests that the formation of specific membranous replication organelles, which allow close spatial coupling of viral RNA replication and virion assembly, is not inhibited by this mutation. At present we can only speculate about the underlying mechanism that is causing the E530A-mediated virion morphogenesis phenotype. One possible scenario could be that the mutant viral RNA is not incorporated into the nucleocapsids or that defective particles are formed that get subsequently misrouted to sites that do not support particle release. Support for the formation of defective particles comes from the sucrose gradient centrifugation of cell lysates electroporated with the mutant viral RNA that revealed an accumulation of slow sedimenting core complexes similar to a p7-QQ mutant with a defect in viral particle production (S3 Fig and [74]). Interestingly, slow sedimenting core complexes similar to the p7-QQ and the NS3 E530A mutant were also observed at early time points in the case of WT- Jc1, suggesting that these slow sedimenting core complexes were part of naturally occurring assembly intermediates that disappeared during that maturation process [74,90]. In view of these observations, it seems possible that in the case of the E530A mutation the viral particles could not complete their maturation. Whether those core complexes represent arrested capsid assembly intermediates or aberrant core aggregates remains to be investigated. However, our analysis is in line with other reports that NS3 also participate in the assembly of large ribonucleoprotein particles such as viral nucleocapsids [33,68]. Importantly, related viral NS3 proteins support virus assembly in different ways. For example, assembly of Kunjin virus, a flavivirus, requires the viral NS3 protein in cis [91]. In contrast, NS3 from yellow fever virus (YFV) can perform its virus assembly function when expressed in trans [92]. Importantly, the helicase activity of YFV NS3 is not required during assembly viral particles. In the case of HCV, mutations in the NS3 helicase domain stimulating viron morphogenesis could be identified, which do not detectably change the NTPase or helicase activities of NS3 [33]. This observation is suggesting that HCV NS3 serves additional functions in infectious virus assembly, independent of its role as helicase protein in HCV RNA replication. For the E530A mutation it is also unlikely that it influences the enzymatic activities of NS3 because it allows wildtype-level viral RNA replication (Figs 3D and 5C). Accordingly, the observed defect in virion morphogenesis caused by E530A (Fig 5D) most likely stems from alterations of some other functions of the NS3 protein which support the assembly of large ribonucleoprotein particles such as viral nucleocapsids. Of note, HCV appears to rely also on cellular helicases such as DDX3X for the assembly of multiple concurrent cellular machineries that are exploited by this virus for its life cycle [93,94].

The identification of surface residue E530 being important for virion morphogenesis is in line with other reports that identified NS3 residues critical for particle formation [33,68]. For instance, a functional rescue of packaging-inhibiting HCV core mutations by a NS3 mutation K272R [NS3 numbering] supports a functional interplay between those two proteins during HCV virion morphogenesis [68]. Interestingly, in the case of the closely-related classical swine fever virus (CSFV) NS3 helicase mutations were selected, which were individually sufficient for the rescue of virion production in a virus mutant with a deletion of the almost the entire core protein [95]. One of these mutations (NS3 E571G) is in the CSFV NS3 at the orthologous position to E530 in HCV NS3 [96]. Absence of core most likely requires recruitment of other proteins such as cytoplasmic RNA chaperones or RNA binding proteins to functionally replace core in the virus particle to allow for viral RNA condensation during packaging. The authors suggested NS3 could play an important role in these recruitments or could be potentially incorporated into these core-defective virus particles [95]. Interestingly, introduction of one of the NS3 helicase rescue mutations into the parental CSFV attenuated virus growth and abrogated the detectable incorporation of core protein into virus particles, which then accumulated intracellularly [95]. Thus, modified NS3 could have the ability to counteract core integration into viral particles, most likely by modulating core-RNA-interactions. Together these finding are further emphasizing the important role of NS3/4A-containing multi-protein complexes in many steps of the HCV life cycle [13,33,37,40]. Of note, NS3 relies on a wide array of host cell factors such as Y-box binding protein (YBX)-1 to regulate the equilibrium of viral RNA translation, RNA replication and RNA genome packaging [97]. Whether or not host factors also bind to the distinct NS3 surface areas described in this work to modulate, in a temporal and spatial fashion, the assembly of dedicated NS3-specific multi-protein complexes during the different steps of the hepaciviral life cycle remains to be investigated. In conclusion, our observations strongly suggest that NS3 surface interactions are critical for the sequential assembly of distinct functional protein complexes and allow NS3 to orchestrate replicase assembly and virion formation.

## Methods

### Cell culture

Huh7 Lunet cells [98], the highly permissive cell line Huh7.5 [99] and Huh7-T7 [100] cells were maintained in Dulbecco’s modified minimal essential medium supplemented with 10% FCS, 100 U penicillin/100 μg/ml streptomycin, and 2 mM L-glutamine. Huh7-T7cells were cultured in the presence of 400 μg/ml G418.

### Plasmids DNAs

HCV genomes Con1[101], JFH1 [102,103] and J6/CF [104] have been described. The subgenomic genotype 2a (JFH1) replicon constructs pFKi389Luc/NS3-3’_dg (designated pFKi 389 FLuc NS3-3’/JFH1), pFKi389Luc/NS2-3’_dg (abbreviated pFKi 389 FLuc NS2-3’/JFH1) and the replication-deficient mutant versions FKi 389 FLuc NS3-3’/JFH1/GND and FKi 389 FLuc NS2-3’/ JFH1/GND have been described [105]. The subgenomic genotype 1b (Con1) replicons pFKi 341 PI-FLuc NS3-3′/Con1/ET (designated FKi 341 PI-FLuc NS3-3’_Con1) and pFKi-I341PI-Luc/NS3-3′/Con1/GND were described [106,107]. The JFH1 and Con1-derived expression constructs pcite NS3-5B/JFH1, pcite NS2-5B/JFH1, pcite NS3-5B/Con1, pcite NS2-5B/Con1 and mutant derivatives thereof have been described [43]. QuikChange mutagenesis of Con1 NS3 was performed on pKS-HindIII-XhoI-NS3-5A/Con1 [43] and the cloning of mutated NS3 derivatives into the pcite NS3-5B/Con1, pcite NS2-5B/Con1 and pFKi 341 PI-FLuc NS3-3’/Con1, respectively, has been performed as described in [43]. The mutagenesis of JFH1 NS3 was performed using either plit-KpnI-NsiI/NS3/JFH1 or plit28-KpnI-NsiI/NS2-NS3/JFH1, respectively, and verified mutations were cloned into pcite-NS3-NS5B/JFH1 or pFKi 389 FLuc NS3-3’/JFH1 via KpnI/NsiI sites as described [43]. To determine viral genome replication and infectivity release, selected NS3 mutations were introduced into monocistronic *Renilla* luciferase HCV reporter virus JcR2a (pFK JcR2a; [70]) from plit28-KpnI-NsiI/NS2-NS3/JFH1 plasmid via *NotI* and *NsiI* restriction sites. The JFH1_HAF-NS2 plasmid encoding the JFH1 genome with a HA-FLAG-tagged NS2 protein (HAF-NS2) that we used for the co-immunoprecipitation experiments has been described [53].

### *In vitro* transcription and electroporation of HCV RNAs

*In vitro* transcripts were generated from HCV bicistronic replicon pFki 389 Fluc NS3-3’/JFH1 or full-length cDNA plasmids JcR2a (pFK JcR2a) after *MluI* linearization. The genotype 1b derived HCV bicistronic replicon derivatives of pFKi 341 PI-Fluc NS3-3’/Con1 were linearized with *ScaI*. All DNA templates were transcribed by T7-RNA polymerase (Ambion, Invitrogen) as described [43,51]. For the electroporation of replicon RNAs into Huh-7 Lunet or full length RNAs into Huh7.5 cells, respectively, cells were resuspended at a concentration of 1.5×10^7^ cells/ml in cytomix buffer supplemented with 2 mM ATP and 5 mM glutathione [108], mixed with RNA before transferring the mixture into 4mm gap cuvette. The following conditions were applied for electroporation: 950 mF and 270 V [101]. After electroporation, cells were immediately transferred to complete medium and seeded as required for the specified assay.

### Luciferase assays to determine RNA replication

Viral RNA replication was determined by a replicon-based reporter assay. At each time point post electroporation (4, 24, 48, and 72 h pe) cells were washed with PBS, detached by scraping into 1 ml of PBS and collected by centrifugation. The cells were lysed in 40 μl of lysis buffer (PJK-GmbH; Kleinblittersdorf, Germany) for the determination of *Firefly* luciferase and 20 μl of the lysate was mixed with 100 μl of Beetle Juice (PJK-GmbH; Kleinblittersdorf, Germany) to determine *Firefly* luciferase activity in the replicon-based replication assay. Measurements were performed with the Junior LB9509 luminometer (Berthold, Berthold Technologies, Freiburg, Germany).

### Luciferase infection assay

Virion morphogenesis was investigated by using the reporter virus system based on pFK JcR2a [70,109]. Quantification of luciferase reporter activity was used to determine HCV genome replication and virus assembly. HCV electroporated Huh7.5 cells were seeded in duplicate into 6-well plates. The transfected cells were harvested 4 h, 24 h, 48 h and 72 h after electroporation (pe). The supernatants of transfected cells were harvested 48 h and 72 h after electroporation and used for reinfection of naïve Huh7.5 cells. After 72 h pi, infected cells were washed with PBS and collected by scraping into 1 ml of PBS followed by centrifugation. Huh7.5 cell pellets were lysed in 40 μl lysis buffer (PJK-GmbH; Kleinblittersdorf, Germany). 20 μl of the lysate was mixed with *Renilla* luciferase assay buffer (15 mM K-phosphate, pH 7.8; 25 mM GlycylGlycine, pH 7.8; 15 mM MgSO_4_; 4 mM EGTA, Coelenterazine 117 μM) and measured in a luminometer (Lumat Junior LB9509; Berthold Technologies, Freiburg, Germany). Intracellular luciferase counts of electroporated cells represent viral genome replication while luciferase values of re-infected cells represent infectivity released by transfected HCV genomes. Each sample was measured in duplicate.

### Determination of cell-associated and released infectious virus particles and HCV core protein amount

Huh7.5 cells were electroporated with 5 μg of the respective JcR2a RNAs and seeded into 6-well plates. To determine the amounts of extracellular infectivity, supernatant was harvested 48 h or 72 h after electroporation, filtered through a 0.45μm-pore-size filter and stored at 4°C. To quantify amounts of intracellular infectivity, cells were rinsed three times with PBS and scraped into 0.5 ml PBS. Cell pellets were obtained by centrifugation for 5 min at 700×g, resuspended in 0.5 ml of complete DMEM and subjected to three freeze-thaw cycles. Debris was removed by centrifugation for 10 min at 20,000 × g. Virus titers were determined by infecting naïve Huh7.5 cells with the respective samples and cell-associated and cell-released infectivity was determined by *Renilla* luciferase assay 72 h pi.

The amounts of core protein were determined as described in [70] with modifications. Briefly, HCV particles within cells were lysed in 1% Triton X-100 containing luciferase lysis buffer. HCV-containing cell culture supernatant were filtered through a 0.45 μm pore-size filter and inactivated with 1 volume of 1% Triton X-100 in PBS. The intra- and extracellular core samples were diluted 20 and 50 times, respectively in Triton X-100 containing PBS with the final concentration of 0.5% Triton X-100.). HCV core protein was quantified using a commercial chemiluminescent microparticle immunoassay (CMIA) (6L47, ARCHITECT HCV Ag Reagent Kit, Abbott Diagnostics, Abbott Park, USA) according to the instructions of the manufacturer.

Virus titers (50% tissue culture infective dose per ml (TCID_50_/ml)) were determined as described elsewhere with slight modifications [106]. Huh7.5 target cells were seeded at a concentration of 1.1×10^4^ cells per well of a 96-well plate in a total volume of 150 μl complete DMEM. Serial dilutions of virus containing supernatant were added with 4 wells per dilution. Three days later, cells were washed with PBS and fixed for 20 min with ice-cold methanol at -20°C. After three washes with PBS NS5A was detected with a 1∶2,000 dilution of antibody 9E10 in PBS for 1 h at room temperature. Cells were washed again three times with PBS and bound primary antibodies were detected by incubation with Cy3–conjugated anti mouse antibody (Dianova), diluted 1∶1,000 in PBS. After 1 h incubation at room temperature cells were washed three times with PBS. Virus titers (TCID_50_/ml) were calculated based on the method of Spearman and Kärber.

### Vaccinia virus infection, DNA transfection and transient protein expression

The experimental setup to perform replication-independent, transient polyprotein expression has been described [43]. Viral polyproteins were expressed from pcite NS3-NS5B encoding HCV nonstructural proteins NS3-NS5B of genotype 1b (Con1) or genotype 2a (JFH1), respectively. Briefly, for SDS-PAGE and Western blot analysis of viral proteins 2 x 10^6^ Huh-7/T7 cells were infected with MVA-T7^pol^ vaccinia virus [110] and subsequently transfected with 8 μg of the respective plasmid DNA by using a PEI transfection protocol. Additional infection of Huh7-T7 cells with MVA-T7 was used to boost the expression of T7 RNA polymerase to allow efficient replication-independent expression of the HCV polyprotein. In the case of IF analysis for protein localization Huh7-Lunet/T7 cells were transfected with 8 μg of the indicated plasmid DNA by Metafectene reagent (Biontex Laboratories GmbH, Munich, Germany) without prior MVA-T7pol vaccinia virus infection.

### SDS-PAGE and western blot

Proteins of Huh7-T7 cell lysates were separated by sodium dodecyl sulfate polyacrylamide gel electrophoresis (SDS-PAGE) using 8% or 10% polyacrylamide-tricine gels [111]. Huh7-T7 cell pellets from a 6-well were lysed by resuspension in 100 μl of cell lysis buffer (Promega, Madison, USA) with phosphatase protein inhibitors (PPI) to preserve NS5A phospho-isoforms. Cell lysates were centrifuged for 1 min at 15,700 g, and the supernatant was mixed with 100 μl of standard SDS-protein sample buffer containing ß-mercaptoethanol. Samples were incubated at 95°C for 10 min to denature proteins, prior to gel loading. After gel electrophoresis, proteins were transferred onto a nitrocellulose membrane (Pall, USA). The membrane was blocked with 5% (w/v) skim milk in phosphate-buffered saline with 0.05% (v/v) Tween 20 (Invitrogen). The following antibodies were used in this study: anti-NS5A 9E10 (a gift from T. Tellinghuisen, Jupiter, FL, USA; [21]), mouse monoclonal antibody against NS3 of the JFH-1 isolate (4D11) (a gift from B. Lindenbach, New Haven; [43]) or anti-NS3 (2E3). Mouse monoclonal antibody recognizing NS3 of the JFH-1 isolate (NS3-2E3) was generated in co-operation with H. Tang, Florida State University, USA [70]. Antibodies were diluted in 2% (w/v) skim milk in phosphate-buffered saline with 0.05% (v/v) Tween 20. Horseradish peroxidase-conjugated species-specific secondary antibodies (Dianova) were applied to detect the primary antibodies. Membranes were washed three times with PBS containing 0.05% (v/v) Tween 20. Western Lightning Chemiluminescence Reagent Plus (Perkin Elmer) was applied to the membrane for 1 min prior to imaging using a LAS 4000 imaging system (Biorad, Munich).

### Co-immunoprecipitation

Huh7.5 cells were transfected with the indicated HCV HA-F-NS2_JFH1 RNA, and samples were harvested 72 h later by scraping into IP buffer (0.5% n-dodecyl-β-D-maltoside, 100 mM NaCl, 20 mM Tris-Cl, pH 7.5). After 60 min incubation on ice, cell debris was removed by 15 min centrifugation at 20,000 x g. Samples were incubated with HA-specific antibody beads (Cell Signaling) over night at 4°C with continuous head-over-tail rotation. After three times washing with IP buffer, samples were eluted into sample buffer and separated by electrophoresis into a 12% Tris-Tricine gel. Proteins were transferred onto PVDF membrane and HCV proteins were detected by Western blot as described above.

### Proteinase K digestion protection assay

The proteinase K digestion protection assay was per- formed as described in [74] with some modifications. Briefly, wild-type or the E530A variant of FK- JcR2a RNA was electroporated into Huh7.5 cells. The cells were collected at 48 h pe in PBS, subsequently resuspended in proteinase K buffer (50mM Tris-Cl, pH 8.0, 10 mM CaCl_2_, 1 mM DTT) and subjected to 5 freeze-thaw cycles. Cell lysates were obtained after centrifugation, divided into three groups: untreated or treated with 50 μg/μl of proteinase K (Roche, Mannheim, Germany) on ice for 1 h, and pretreated with 5% (vol/vol) Triton X-100 before proteinase K treatment on ice for 1 h. Proteinase K was then inactivated with 5 mM phenylmethylsulfonyl fluoride (PMSF) on ice for 10 min and cell lysates were mixed with sample buffer. The amount of core protein was determined by Western blot.

### Rate zonal centrifugation assay

The rate zonal centrifugation assay has been described elsewhere [112] was used with slight modifications. Briefly, 4x10^6^ Huh7.5 cells were electroporated with 10 μg in vitro-transcribed HCV RNA JcR2a derivatives and seeded into 10-cm-diameter dishes. At 48 h post-electroporation, cells were washed three times with PBS, trypsinized, pelleted, rinsed two times with DMEM, and resuspended into 0.5 ml PBS. Thereafter, cells were subjected to five repetitive cycles of freezing and thawing in liquid nitrogen and a 37°C heat block. Cell lysates were centrifuged at 20,000 × g for 10 min at 4°C. Supernatants were then collected and directly layered on top of continuous 0 to 30% sucrose-PBS gradients and centrifuged at 270,000 × g for 1 h at 4°C using an SW60 Ti rotor (Beckman Coulter, Inc.). Twelve fractions were collected gradually from the top to bottom of the gradients. The density of each fraction and the core protein amount containing therein were determined by refractive index measurement using a refractometer (Kruess, AGS Scientific) and a core CMIA, respectively.

### Immunofluorescence analysis

Cells after electroporation with HCV RNA were seeded on 35 mm glass bottom imaging dishes (MatTek Corporation). At 48 h pe, cells were washed and cultured in phenol red-free DMEM at 37°C and 5% CO_2_ in the humidified incubation chamber of the imaging system. Live-cell confocal imaging was performed with the spinning disc confocal microscope PerkinElmer UltraVIEW Vox Spinning Disc CSU-X1 equipped with Nikon TiE, the EM-CCD Hamamatsu ImageEM X2 camera, and an automated Nikon perfect focus system as previously described [29]. The lipid droplets were stained with LipidTox Deep Red (Thermofisher, catalog number H34477).

## Supporting information

S1 Fig The analyzed NS3 protease surface mutations do not inhibit NS2 autoprotease activation by NS3. The MVA/T7^pol^ expression system was used to interrogate the NS3 protease surface mutations for effects on NS2 autoprotease activation by NS3 and HCV polyprotein processing. NS3 mutations were introduced into pcite-NS2-3’/JFH1 or pcite-NS2-3’/Con1 plasmids, respectively and plasmids were transfected into Huh-7/T7 cells infected with MVA-T7pol vaccinia virus. (A and C) Schematic representation of the pcite-NS2-3’/JFH1 or pcite-NS2-3’/Con1 expression plasmids. (B and D) Western blot analysis of HCV NS2-5B polyprotein processing and NS5A hyperphosphorylation are shown for the NS2-5B polyprotein of genotype 2a and genotype 1b, respectively. Positions of NS2-4A and NS2-3 precursor proteins as well as NS2, NS3 and NS5A phospho-isoforms (p56, basal and p58, hyperphosphorylated) are indicated by arrows. Western blots shown are representative for three independent experiments.
(TIF)

S2 Fig Impact of NS3 surface mutations on the HCV genome replication and infectivity release. The indicated NS3 mutations were introduced into the full-length HCV JcR2a. (A) Determination of viral genome replication. Huh7.5 cells were electroporated with in vitro transcribed viral RNA and kinetics of HCV genome replication were quantified 4, 48 and 72 hours post electroporation by measuring *Renilla* luciferase activity (relative light units, RLU). (B) Analysis of virion morphogenesis. Supernatants containing released infectious particles were harvested 48 and 72 hours post electroporation and used for inoculation of naïve Huh7.5 cells. Three days after infection, cells were harvested, cell lysates were prepared, and intracellular luciferase activity was determined. The background of the luciferase assay is indicated by the horizontal dashed line. (C) Virus amounts contained in culture supernatants were quantified by limiting dilution assay. Mean and SEM of three independent experiments are shown. Background of the assay is indicated. n.d., not detected.
(TIF)

S3 Fig Sucrose gradient separation of core protein complexes indicate the formation of aberrant non-functional core structures. Postnuclear supernatants of cell lysates obtained by repetitive cycles of freeze and thaw 48 h post-electroporation of Huh7.5 cells with the indicated JcR2a derivatives were layered on top of a preformed continuous 0–30% sucrose density gradient and subjected to centrifugation for 1 h at 270,000 x g. Core content was measured along the gradient by CMIA and normalized to the total core amount in the lysate. Fractions 6 (refraction index, 1.04) and 9 (refraction index, 1.08) are highlighted with black arrows.
(TIF)

S4 Fig The NS3 E530 amino acid substitutions do not detectably affect interaction between NS2, NS3, NS5A and core in NS2 pull-down experiments. (A) Schematic diagram of the full-length JFH1_HAF-NS2 genome used for NS2 co-immunoprecipitation experiments. The HCV proteins are represented as brown boxes. The HA-FLAG epitope is shown as red box. The NS3 E530 mutations are represented as black star. (B) Experimental set-up: Huh7.5 cells were electroporated with WT or mutated RNAs, harvested at 72 h pe and lysed. Mock-transfected cells were used as negative controls. (C) Protein lysates were used for HA-specific immunoprecipitation. Pull down efficiency of HAF-NS2 protein as well as the co-immunoprecipitated NS3, NS5A and core proteins were analyzed by Western Blot. Input lysate and sample containing immunoprecipitated proteins were loaded on the gel in the ration 1:10.
(TIF)

S5 Fig NS3 E530A and E530G mutations allowed nonstructural protein-induced E2 redistribution. The HCV_TCP_ system was used to determine the distribution and dynamics of NS5A and E2 in the context of NS3 mutations (E530A, E530G) when compared to WT NS3 by live-cell imaging and CLEM. Huh7-Lunet /CD81H cells stably expressing C-NS2/^egfp-CS^E2 were electroporated with *in vitro* transcribed RNA of HCV sub-genomic replicon sgJFH1(NS3-NS5B)NS5A-mCherry WT, NS3-E530A or NS3-E530G, respectively. Cells were subjected to confocal live-cell imaging microscopy to monitor HCV E2 and NS5A signals. Extracted time frames were analysed at 48 h pe. Scale bar: 5 μm. Cyan arrowheads: E2-NS5A foci. The lipid droplets were stained with LipidTox Deep Red (Thermofisher, catalog number H34477).
(TIF)

S1 Text Supporting Text. Supporting Reference list.
(DOCX)
