# Supplementary figures and images for: Characterization of a multipurpose NS3 surface patch coordinating HCV replicase assembly and virion morphogenesis
Source: PLoS Pathog. 2022 Oct 10;18(10):e1010895. doi: 10.1371/journal.ppat.1010895 (PMC9616216; doi:10.1371/journal.ppat.1010895)

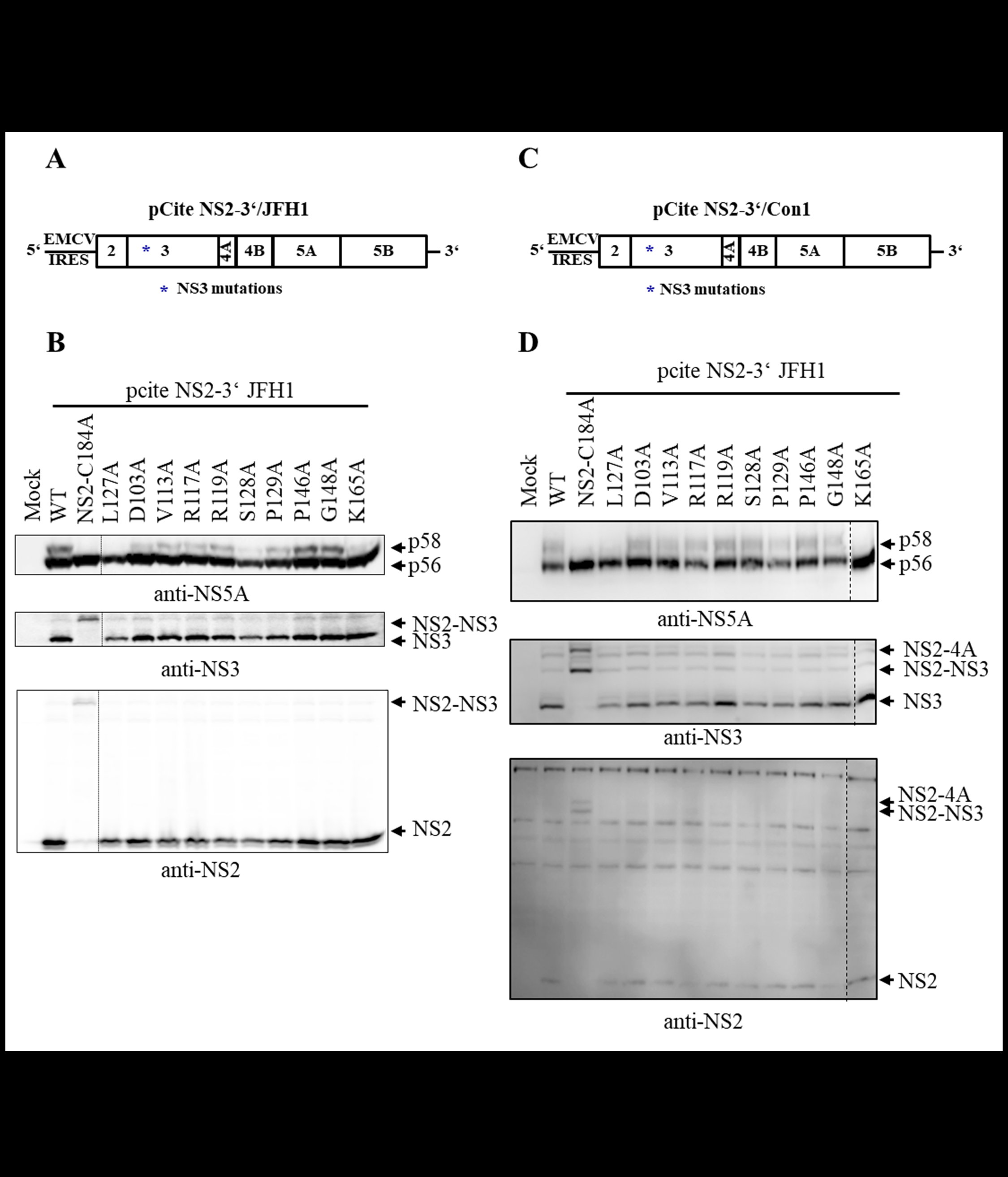

Supplement: S1 Fig — The MVA/T7pol expression system was used to interrogate the NS3 protease surface mutations for effects on NS2 autoprotease activation by NS3 and HCV polyprotein processing. NS3 mutations were introduced into pcite-NS2-3’/JFH1 or pcite-NS2-3’/Con1 plasmids, respectively and plasmids were transfected into Huh-7/T7 cells infected with MVA-T7pol vaccinia virus. (A and C) Schematic representation of the pcite-NS2-3’/JFH1 or pcite-NS2-3’/Con1 expression plasmids. (B and D) Western blot analysis of HCV NS2-5B polyprotein processing and NS5A hyperphosphorylation are shown for the NS2-5B polyprotein of genotype 2a and genotype 1b, respectively. Positions of NS2-4A and NS2-3 precursor proteins as well as NS2, NS3 and NS5A phospho-isoforms (p56, basal and p58, hyperphosphorylated) are indicated by arrows. Western blots shown are representative for three independent experiments. (TIF) [file ppat.1010895.s001.tif]

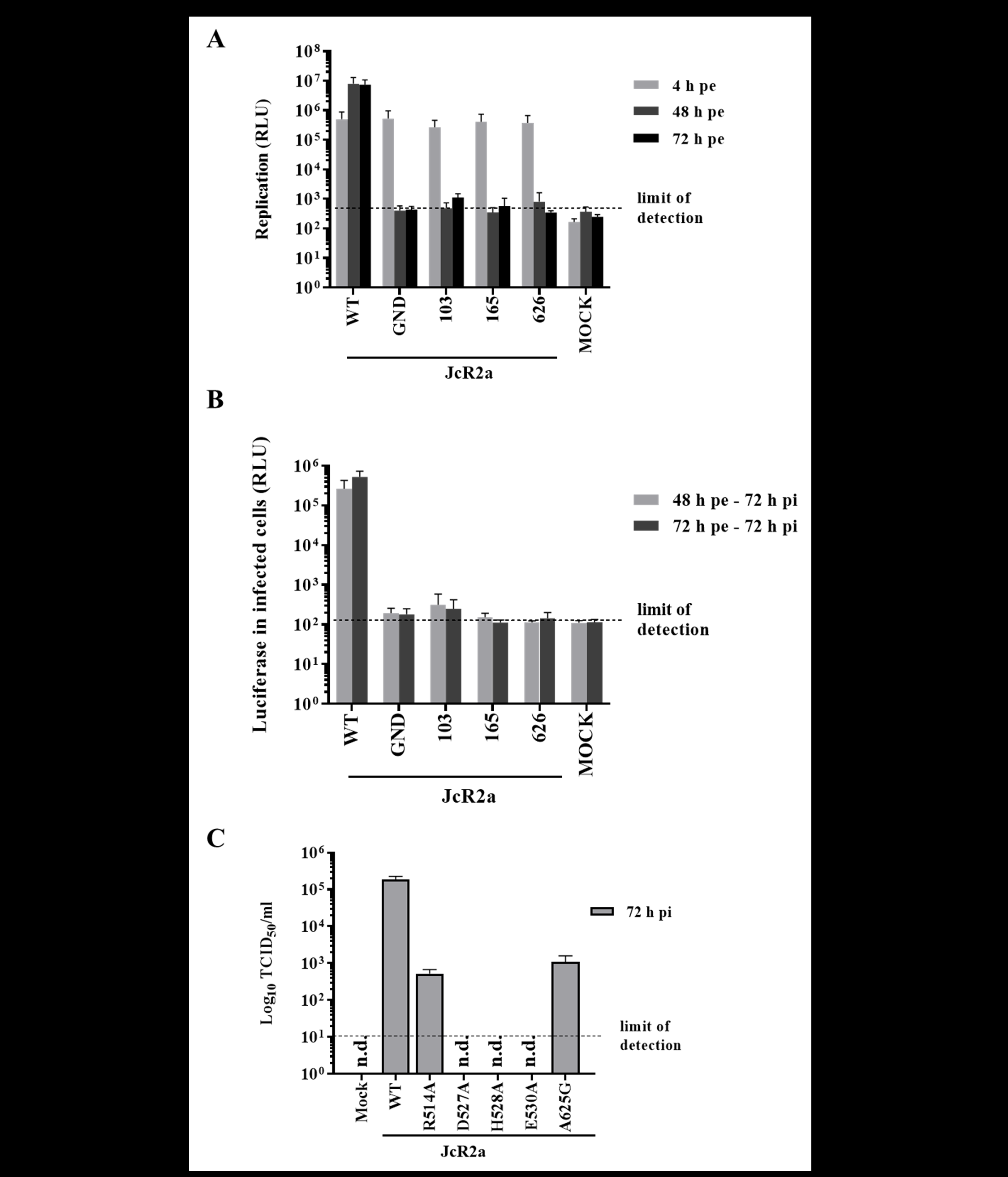

Supplement: S2 Fig — The indicated NS3 mutations were introduced into the full-length HCV JcR2a. (A) Determination of viral genome replication. Huh7.5 cells were electroporated with in vitro transcribed viral RNA and kinetics of HCV genome replication were quantified 4, 48 and 72 hours post electroporation by measuring Renilla luciferase activity (relative light units, RLU). (B) Analysis of virion morphogenesis. Supernatants containing released infectious particles were harvested 48 and 72 hours post electroporation and used for inoculation of naïve Huh7.5 cells. Three days after infection, cells were harvested, cell lysates were prepared, and intracellular luciferase activity was determined. The background of the luciferase assay is indicated by the horizontal dashed line. (C) Virus amounts contained in culture supernatants were quantified by limiting dilution assay. Mean and SEM of three independent experiments are shown. Background of the assay is indicated. n.d., not detected. (TIF) [file ppat.1010895.s002.tif]

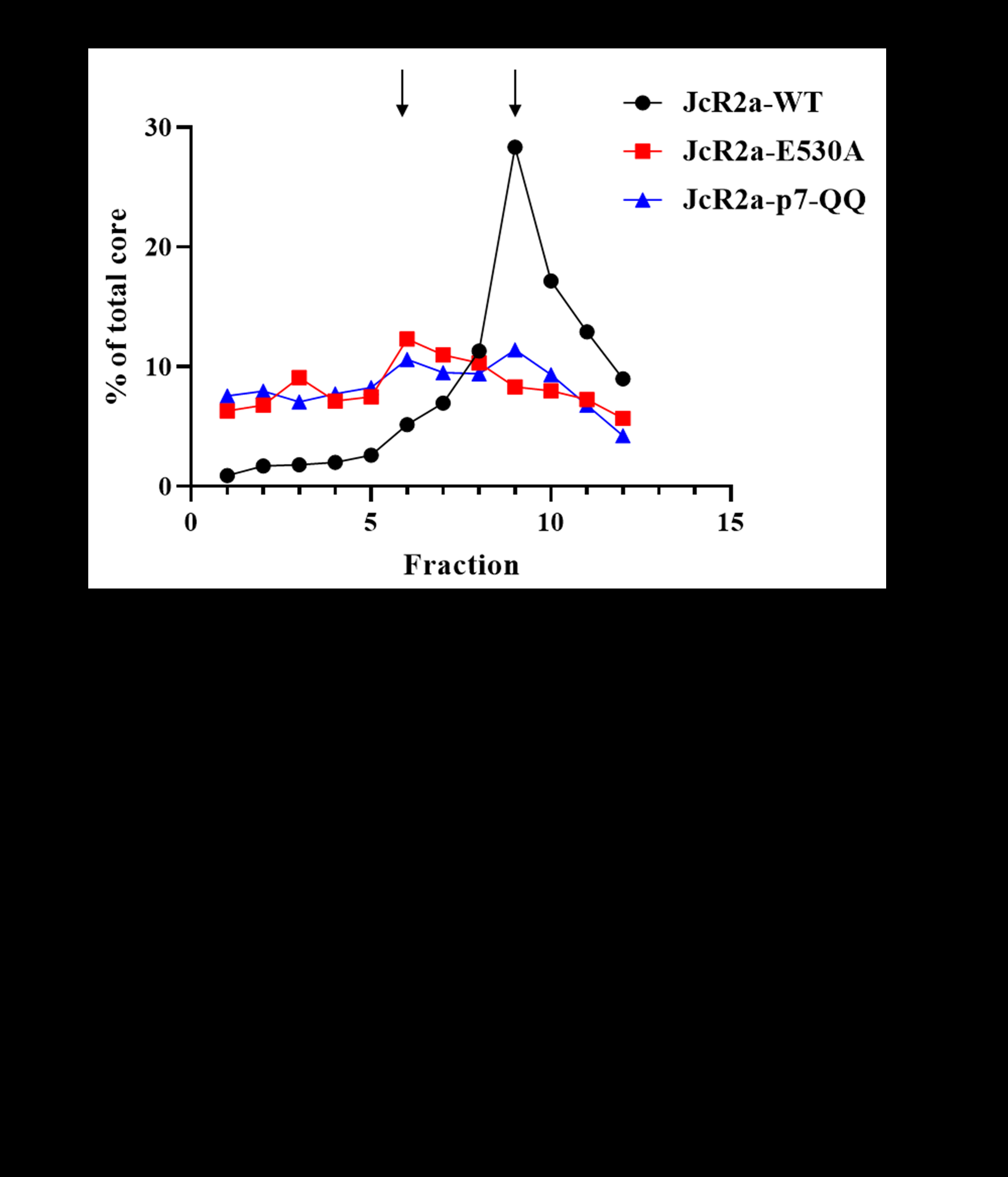

Supplement: S3 Fig — Postnuclear supernatants of cell lysates obtained by repetitive cycles of freeze and thaw 48 h post-electroporation of Huh7.5 cells with the indicated JcR2a derivatives were layered on top of a preformed continuous 0–30% sucrose density gradient and subjected to centrifugation for 1 h at 270,000 x g. Core content was measured along the gradient by CMIA and normalized to the total core amount in the lysate. Fractions 6 (refraction index, 1.04) and 9 (refraction index, 1.08) are highlighted with black arrows. (TIF) [file ppat.1010895.s003.tif]

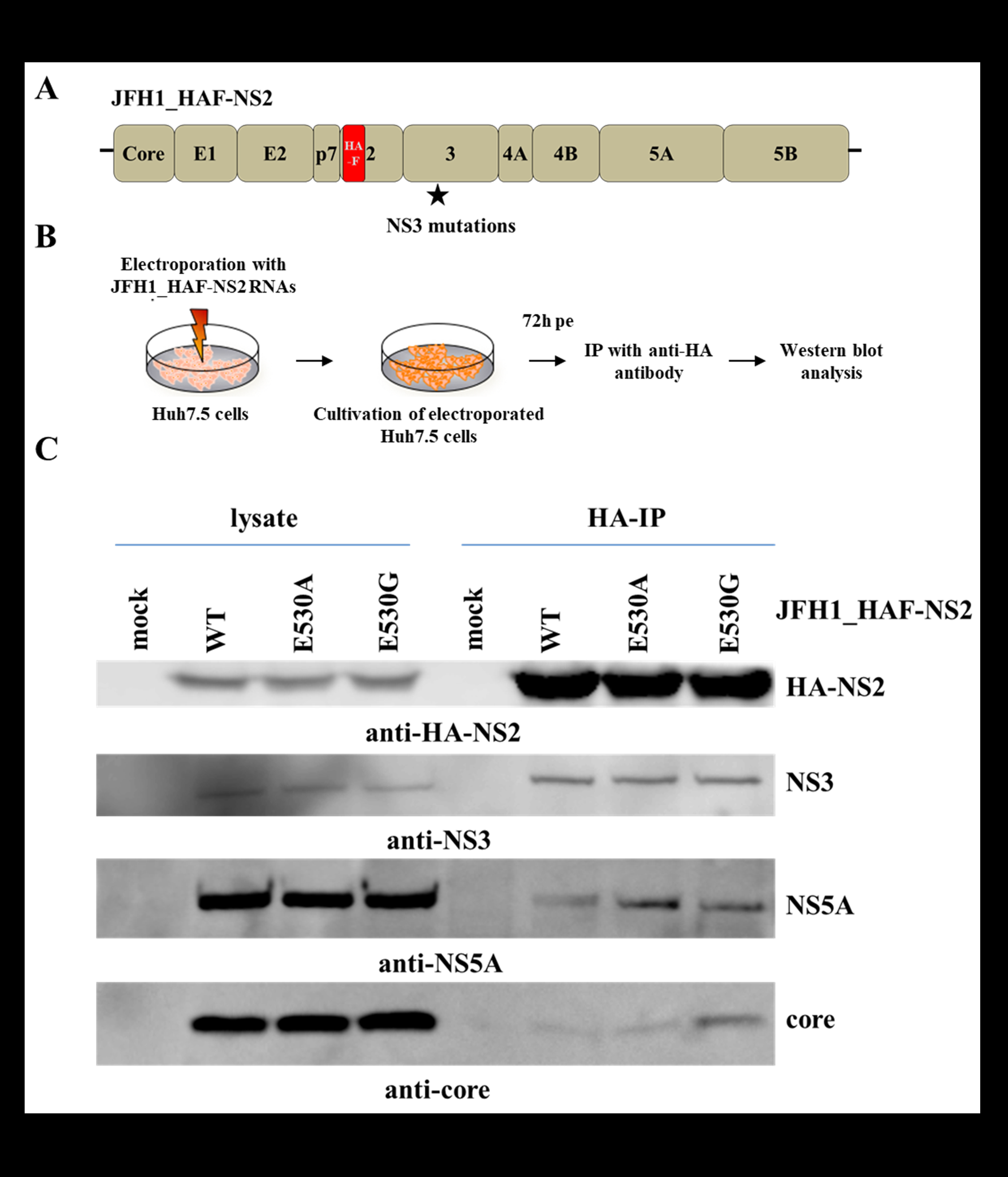

Supplement: S4 Fig — (A) Schematic diagram of the full-length JFH1_HAF-NS2 genome used for NS2 co-immunoprecipitation experiments. The HCV proteins are represented as brown boxes. The HA-FLAG epitope is shown as red box. The NS3 E530 mutations are represented as black star. (B) Experimental set-up: Huh7.5 cells were electroporated with WT or mutated RNAs, harvested at 72 h pe and lysed. Mock-transfected cells were used as negative controls. (C) Protein lysates were used for HA-specific immunoprecipitation. Pull down efficiency of HAF-NS2 protein as well as the co-immunoprecipitated NS3, NS5A and core proteins were analyzed by Western Blot. Input lysate and sample containing immunoprecipitated proteins were loaded on the gel in the ration 1:10. (TIF) [file ppat.1010895.s004.tif]

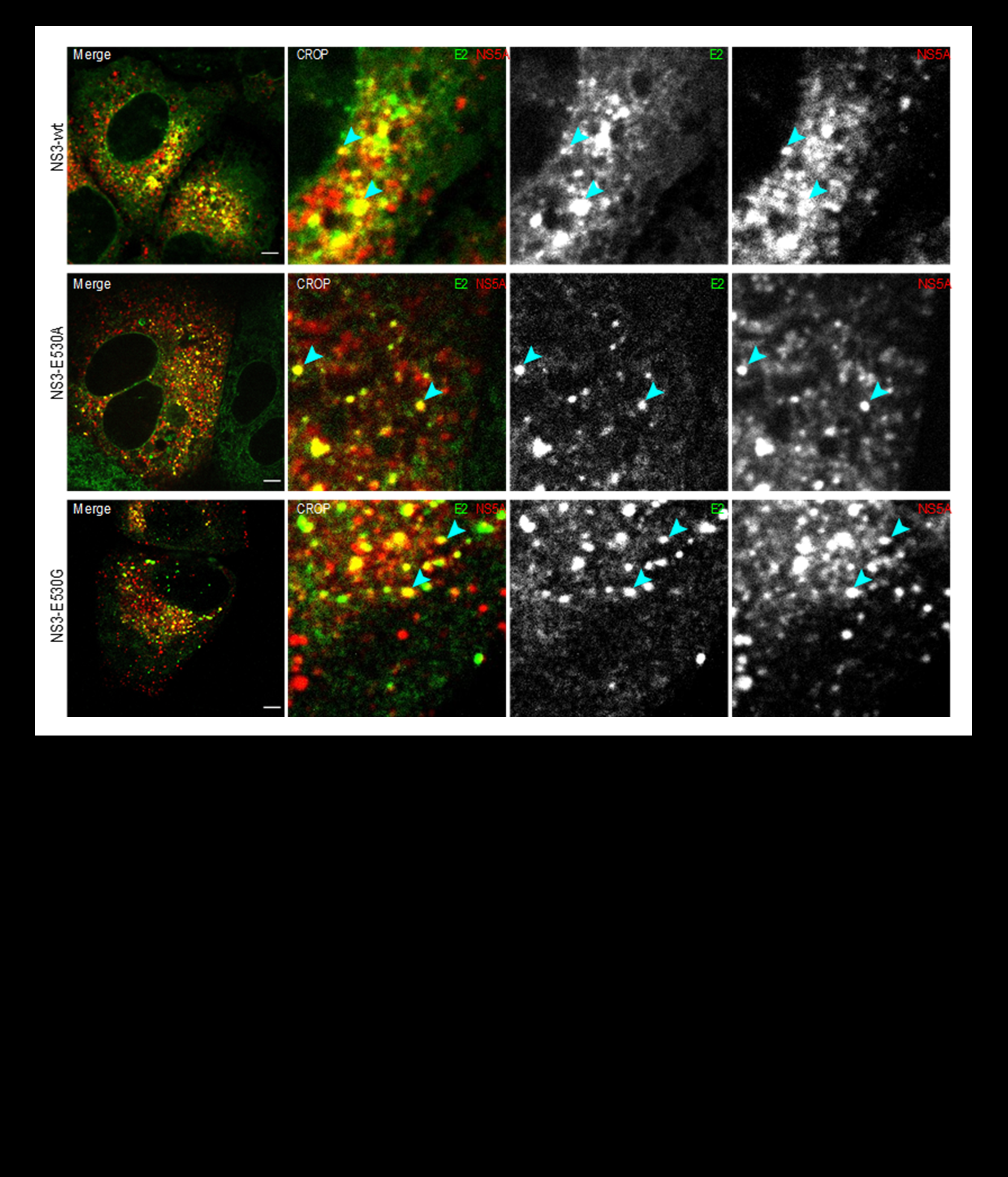

Supplement: S5 Fig — The HCVTCP system was used to determine the distribution and dynamics of NS5A and E2 in the context of NS3 mutations (E530A, E530G) when compared to WT NS3 by live-cell imaging and CLEM. Huh7-Lunet /CD81H cells stably expressing C-NS2/egfp-CSE2 were electroporated with in vitro transcribed RNA of HCV sub-genomic replicon sgJFH1(NS3-NS5B)NS5A-mCherry WT, NS3-E530A or NS3-E530G, respectively. Cells were subjected to confocal live-cell imaging microscopy to monitor HCV E2 and NS5A signals. Extracted time frames were analysed at 48 h pe. Scale bar: 5 μm. Cyan arrowheads: E2-NS5A foci. The lipid droplets were stained with LipidTox Deep Red (Thermofisher, catalog number H34477). (TIF) [file ppat.1010895.s005.tif]
