# Supplementary material for: Characterization of a multipurpose NS3 surface patch coordinating HCV replicase assembly and virion morphogenesis
Source: PLoS Pathog. 2022 Oct 10;18(10):e1010895. doi: 10.1371/journal.ppat.1010895 (PMC9616216; doi:10.1371/journal.ppat.1010895)
Supplement: S1 Text — Supporting Reference list. (DOCX) [file ppat.1010895.s006.docx]

**S1 Text**

**E530A is not disrupting NS2-NS3 interactions essential for the formation of NS2-containing complexes involved in initiation stages of virion morphogenesis**. Assembly defects caused by NS2 mutations could be compensated by pseudoreversions in NS3 indicating the interaction between NS2 and NS3 is critical for virion morphogenesis [1]. Since the NS3 E530A and E530G mutations specifically abolished virus assembly, we tested if this mutation affects NS2 and NS3 interaction by NS2 pull-down experiments. For these purposes, we used a fully functional JFH1-derivative (JFH1_HAF-NS2) with an N-terminally HA-FLAG-tagged NS2 variant (HAF-NS2; [1]) suitable for efficient HA-specific immunoprecipitation (S4A Fig). Accordingly, we introduced E530A and E530G mutations into JFH1_HAF-NS2, generating JFH1_HAF-NS2/E530A and JFH1_HAF-NS2/E530G, respectively. JFH1_HAF-NS2/wt, JFH1_HAF-NS2/E530A and JFH1_HAF-NS2/E530G RNAs were electroporated into Huh7.5 cells and lysates from electroporated cells were used for immunoprecipitation with an HA-antibody (S4B Fig). We analyzed HAF-NS2-specific immunocomplexes by Western blot for differences in co-precipitation of HAF-NS2 with core, NS3 and NS5A, respectively (S4C Fig). Western blot analysis of cell lysates showed that comparable amounts of HAF-NS2, core, NS3 and NS5A proteins were produced by cells electroporated by either of the three analyzed RNAs (S4C Fig). After HAF-NS2 immunoprecipitation, we detected comparable co-precipitation of NS3, core and NS5A with HAF-NS2 for WT and the 2 mutants. These results suggest that the observed lost-of-function phenotype for E530A and E530G mutations is not caused by the loss of interactions between these viral proteins.

**Formation of E2-NS5A Double-Positive Structures in Close Proximity to Lipid Droplets is not blocked by packaging-inhibiting NS3 E530 mutations**

It has been established that assembly-dependent subcellular relocalization of E2 from “regular” ER, where it is retained because of ER retention signals [2, 3], to “specific” ER membranes wrapping LDs and containing in addition NS5A, a marker for the viral replicase complex [1, 4], requires replicating HCV RNA [5]. Since this E2 relocalization is NS2-dependent but core-independent, it is consistent with the model that NS2 orchestrates HCV assembly by interacting with p7 and E1/E2 and, via interaction with NS3, recruits the replicase. To investigate if the packaging-inhibiting NS3 mutations block the formation of E2-NS5A double-positive structures in close proximity to lipid droplets in cells producing HCV-like Particles, we used the HCV_TCP_ system (Huh7-Lunet /CD81H cells stably expressing C-NS2/^egfp-CS^E2) and HCV sub-genomic replicon sgJFH1(NS3-NS5B)NS5A-mCherry WT, NS3-E530A or NS3-E530G, respectively. After electroporation with replicon RNA we determine the distribution and dynamics of NS5A and E2 in the context of NS3 mutations (E530A, E530G) and compared the results to WT NS3 by live-cell imaging and CLEM. However, we did not observe striking differences that could explain the observed virion morphogenesis defect caused by the NS3 E530A mutation (S5 Fig).

**References**

1. Jirasko V, Montserret R, Lee JY, Gouttenoire J, Moradpour D, Penin F, et al. Structural and functional studies of nonstructural protein 2 of the hepatitis C virus reveal its key role as organizer of virion assembly. PLoS pathogens. 2010;6(12):e1001233. doi: 10.1371/journal.ppat.1001233. PubMed PMID: 21187906; PubMed Central PMCID: PMC3002993.

2. Duvet S, Cocquerel L, Pillez A, Cacan R, Verbert A, Moradpour D, et al. Hepatitis C virus glycoprotein complex localization in the endoplasmic reticulum involves a determinant for retention and not retrieval. The Journal of biological chemistry. 1998;273(48):32088-95. doi: 10.1074/jbc.273.48.32088. PubMed PMID: 9822684.

3. Cocquerel L, Meunier JC, Pillez A, Wychowski C, Dubuisson J. A retention signal necessary and sufficient for endoplasmic reticulum localization maps to the transmembrane domain of hepatitis C virus glycoprotein E2. J Virol. 1998;72(3):2183-91. doi: 10.1128/JVI.72.3.2183-2191.1998. PubMed PMID: 9499075; PubMed Central PMCID: PMCPMC109514.

4. Miyanari Y, Atsuzawa K, Usuda N, Watashi K, Hishiki T, Zayas M, et al. The lipid droplet is an important organelle for hepatitis C virus production. Nature cell biology. 2007;9(9):1089-97. Epub 2007/08/28. doi: ncb1631 [pii]

5. Lee JY, Cortese M, Haselmann U, Tabata K, Romero-Brey I, Funaya C, et al. Spatiotemporal Coupling of the Hepatitis C Virus Replication Cycle by Creating a Lipid Droplet- Proximal Membranous Replication Compartment. Cell Rep. 2019;27(12):3602-17 e5. doi: 10.1016/j.celrep.2019.05.063. PubMed PMID: 31216478.
